# Exploring genetic diversity of potential legume, *Vigna angularis* (Willd.) Ohwi and Ohashi through agro-morphological traits and SSR markers analysis

**DOI:** 10.1371/journal.pone.0312845

**Published:** 2024-12-06

**Authors:** Deepika Deshahalli Divakara, Padmavati Ganpat Gore, Kuldeep Tripathi, Ashvinkumar Katral, Debjani Roy Choudhury, Golsar Jagadeesh Abhishek, Shridhar Ragi, Danakumar Thippeswamy, Vignesh Muthusamy, Dinesh Kumar Sharma, Rakesh Singh, Kailash Chandra Bhatt

**Affiliations:** 1 The Graduate School, ICAR-Indian Agricultural Research Institute, New Delhi, India; 2 ICAR-National Bureau of Plant Genetic Resource, Pusa, New Delhi, India; 3 ICAR-Indian Agricultural Research Institute, Pusa, New Delhi, India; ICAR - Central Tobacco Research Institute, INDIA

## Abstract

Adzuki bean, an underutilized grain legume, has a significant potential for enhancing food and nutritional security. The main obstacles to developing new cultivars and promoting the adzuki bean as a mainstream pulse crop are a lack of awareness about its potential and insufficient information on crop its genetic diversity. Here, we aimed to explore the untapped potential of adzuki bean germplasm by evaluating its agro-morphological traits and diversity at the molecular level and also to identify trait-specific germplasm by utilizing 100 adzuki bean accessions conserved in the Indian National Genebank. Significant variations was recorded for the morphological traits and identified promising accessions exhibiting desirable traits, such as early flowering (IC341945, EC340257 and EC340283), number of primary branches (IC341945 and IC469175), number of clusters per plant (EC000264, IC167611 and IC341939), number of pods per plant (IC469175, EC34264, EC000264), early maturity (EC340283; EC120460; IC341941) and number of seeds per pod (EC340240, IC455396 and IC341955). Molecular characterization of diverse accessions using 22 polymorphic SSR markers identified a total of 50 alleles, with a mean of 2.27 alleles per loci. The polymorphic information content (PIC) ranged from 0.03 to 0.46, indicating informativeness of markers in distinguishing diverse accessions. Further, the gene diversity among the accessions ranged from 0.03 to 0.57 with a mean of 0.19. Population structure analysis grouped the accessions into three genetic groups, supported by Principal Coordinate Analysis (PCoA) and a phylogenetic tree. Additionally, Analysis of Molecular Variance (AMOVA) confirmed a substantial genetic diversity among the adzuki bean accessions. Thus, the combined assessment of agro-morphological traits and molecular markers effectively distinguished adzuki bean accessions and provided valuable insights in understanding untapped variation at both morphological and molecular levels. The promising accessions identified in the study hold potential for integration into legume improvement programs through introgression breeding, contributing to the development of adzuki bean varieties with target trait.

## Introduction

Adzuki bean, [*Vigna angularis* (Willd) Ohwi and Ohashi], is a member of the family Fabaceae, with a chromosome number of 2n = 2x = 22 and a genome size of 538 mb [1]. *Vigna angularis* var. *nipponensis* (Ohwi) Ohwi and H. Ohashi is a progenitor of the adzuki bean, found in parts of the North Eastern hilly regions of India [2–4]. Adzuki bean is considered one of the twelve most important grain legume crops worldwide and is an essential protein-rich legume, contributing significantly to food and nutritional security. The major adzuki bean producers are China and Japan, followed by Korea, New Zealand, Taiwan, the Philippines, India, and Thailand [5]. In India, it is primarily grown in the North Eastern and North Western hill zones [6].

Adzuki bean offers a versatile nutritional profile, with their young pods serving as vegetables, dry beans as grains, and foliage as livestock fodder and are well-suited for multiple cropping systems alongside rice and maize [7]. Renowned as the ’red pearl’ of beans and the ’weight loss bean’, adzuki beans are recognized for their low calorie and low-fat content, high digestible protein, and abundant bioactive compounds [8, 9]. Additionally, adzuki bean fix atmospheric nitrogen, thereby improving soil fertility. They can also be grown as a cover crop, preventing soil erosion and improving soil structures [10]. This makes them a promising legume crop for addressing food and nutrient security, especially among economically disadvantaged and marginalized populations in temperate regions and the humid subtropic to temperate region of the northeastern hilly region of India.

Scientists and nutrition experts predict that the post-pandemic landscape will necessitate food scientists, breeders, and crop bioengineers to adjust their strategies towards locally adapted food systems [11]. Adzuki beans, rich in protein, dietary fibres, vitamins, minerals (magnesium, potassium, iron, zinc) and antioxidants, boosts immunity, reduce obesity, and improve kidney health [12, 13], making them crucial for dietary diversification and resilience against malnutrition in a global pandemic. To promote their widespread cultivation, it is crucial to encourage the development of new and improved varieties. Recognizing the germplasm as a foundational element, there is a pressing need for the thorough characterization and evaluation of genetic resources to understand the extent of diversity available for crop improvement. This step is crucial to harness the full potential of adzuki beans in addressing nutritional and food security challenges.

Assessing germplasm effectively involves a comprehensive approach that combines agro-morphological and molecular studies. Morphological traits are commonly employed as parameters, offering valuable insights into quantifying genetic variation and measuring their performance under appropriate growing environments [14, 15]. With the advancements in molecular technology, the characterization of germplasm has gained momentum, allowing the generation of genotypic variation at the DNA level. The integration of DNA profile data with statistical techniques facilitates the calculation of genetic diversity. Various molecular markers, such as RFLPs, RAPDs, AFLPs, SSRs, ISSRs, and SNPs, are utilized in genetic diversity studies across different crops. Notably, simple sequence repeats (SSRs) markers are preferred due to their co-dominant nature, abundance, and reproducibility. In the evaluation of genetic diversity within *Vigna* species, SSRs have been widely employed [16, 17]. Therefore, the present research program aims to assess the genetic diversity of adzuki bean accessions by employing a combination of agro-morphological traits and SSR markers. This comprehensive approach ensures a thorough understanding of the genetic landscape of adzuki beans, contributing valuable insights for their improvement and cultivation.

## Materials and methods

### Plant materials

A diverse set of 100, exotic and indigenous germplasm of adzuki bean conserved in the National Genebank of India, at ICAR-National Bureau of Plant Genetic Resources, New Delhi, was used in the present investigation along with three checks (HPU-51, Totru Local, and Grams Local2). The list of all the accessions along with geographical location is given in S1 Table. All the accessions were grown in experimental fields of ICAR-National Bureau of Plant Genetic Resources (ICAR-NBPGR), Regional Station Shimla, Himachal Pradesh, India. This regional station is located at 31˚10’ N latitude, 77˚17’ E longitude, 2276 meters above mean sea level (AMSL), receiving annual rainfall of 1145 mm. The area falls within a humid temperate agroclimatic condition zone. The cultivation spanned two consecutive years, during the kharif seasons of 2021 and 2022, in Augmented Block Design (ABD) with seven blocks, and the checks were randomized within each block (S1 Fig). Each row measured 4 meters, with a spacing of 60 cm between rows, and 20 seeds were sown per accession. All the standard agronomic practices were followed to raise the crop.

### Phenotypic observations

Data on 22 agro-morphological traits including 10 quantitative and 12 qualitative were recorded using descriptors developed by ICAR-NBPGR and Bioversity International (1980) [18]. The quantitative traits were recorded as follows:—days to 50% flowering (DFF), number of primary branches (PB), number of clusters per plant (CLP), number of pods per cluster (PPC), number of pods per plant (PPP), plant height (PH in cm), days to 80% maturity (DM), seed yield per plant (SYPP in g), 100 seed weight (SW in g) and number of seeds per pod (SPP). Twelve qualitative traits which consist of early plant vigour, plant habit, plant growth habit, leaf colour, leaf surface, leaflet shape, stem surface, flower colour, stem colour, pod angle, pod surface and seed coat colour were also recorded (S2 Table).

### DNA isolation

DNA isolation was carried out by the Cetyl trimethylammonium bromide (CTAB) method as per Doyle and Doyle, (1990) [19]. DNA quality and purity were tested by spectrophotometer (Nanodrop™, Thermo-Fisher, USA) and DNA quantification was confirmed on 0.2% agarose gel electrophoresis. Finally, the DNA concentration of all the accessions was adjusted to 10 ng/μL for PCR reaction.

### SSR markers and Polymerase Chain Reaction (PCR) amplification

Initially, 75 SSR markers were screened on selected 24 accessions to identify polymorphic markers [16, 17, 20, 21]. After initial screening, 22 SSR markers showed polymorphisms and were used for detailed study in 100 accessions of adzuki bean. The PCR reaction was set for 15 μl of a total volume containing PCR mix (7.5 μl), nuclease-free water (4.5 μl), primer (F+R) (1 μl), and 10 ng/μl template DNA (2 μl) with minor modifications [17]. The details of the standardized program for amplification were as follows: initial denaturation was carried out for 4 min at 94˚C, followed by 36 cycles of denaturation for 30 sec at 94˚C, annealing for 30 sec at 48–55˚C temperature (standardization of annealing temperature was carried out by gradient PCR for each SSR marker), extension for 30 sec at 72˚C and final extension for 7 min at 72˚C. The amplified product was separated on 3% metaphor agarose (Lonza, USA) gel for 3h at 110 V and gel images were captured by using a gel documentation system (Alpha Imager1, USA).

### Data scoring and statistical analyses

The quantitative traits data of both years were separately taken for calculating ANOVA at *p* < 0.05 level, descriptive statistics viz., mean, range, standard deviation, standard error, coefficient of variation (%). Agro-morphological quantitative trait data of both seasons were combined for calculating genetic parameters like phenotypic coefficient of variation (PCV), genotypic coefficient of variation (GCV), heritability in a broad sense (H^2^) and genetic advance percent of means (GAM) using the “Trait-Stats” package implemented in Rv4.2.3. Multivariate analysis including principal component analysis, and hierarchical cluster analysis were performed. Correlation among the quantitative traits was performed using the “GGally” package implemented in Rv4.2.3. Further, scree plot, principal component analysis (PCA) and cluster diagrams (k-means clustering) were deduced using “factoextra”, and “FactoMineR” packages implemented in Rv4.2.3.

Among the 100 accessions of adzuki bean, amplified products of 22 loci were scored manually with a 100 bp DNA ladder (G-Biosciences, USA). The genetic diversity indices such as major allele frequency (MAF), observed heterozygosity (Ho), number of alleles, genetic diversity (GD), and polymorphic information content (PIC) of all the SSR loci were calculated using Power Marker v3.25 [22]. Genetic distances among the adzuki bean accessions and the Neighbour-joining (NJ) tree were constructed using Power Marker v.3.25. Using STRUCTURE v.2.3.4 software which works on the Bayesian model, the population structure of adzuki bean accessions was determined [23]. Clusters number (k) ranged between 2 to 10 and five replications were run for each k value analysis. Burn-in and Markov chain Monte Carlo were 1,00,000 generations of each run in STRUCTURE v.2.3.4. After obtaining LnP (D) for each K, the values of ΔK were derived [24]. Utilising the STRUCTURE harvester, the final population count for the investigated accessions was determined. Analysis for molecular variation (AMOVA) and Principal Coordinate Analysis (PCoA) were computed for the data set using GenAlEx V6.5 software.

## Results

### Quantitative traits exploration

Analysis of variance revealed that highly significant variation was present between and within accessions of studied adzuki bean at *P* < 0.01 level, except number of primary branches and number of pods per cluster, which showed significant variations at *p* < 0.05 level (Table 1).

**Table 1:** Augmented ANOVA for 10 quantitative traits of adzuki bean for season 1 (2021) and season 2 (2022).

Source of variation	The mean square values for Season 1						The mean square values for Season 2				
	Germplasm	Germplasm: Check	Treatment: Test vs. Check	Treatment: Test	Block	Germplasm		Germplasm: Check	Treatment: Test vs. Check	Treatment: Test	Block
**DF**	102	2	99	1	6	102		2	99	1	6
**DFF**	35.59 **	63.05**	34.06**	132.3**	0.94	38.97**		97.76**	35.4**	267.86**	5.91**
**PB**	0.46*	1.07*	0.43*	1.87**	0.21	0.47*		0.67*	0.46*	1.47*	0.23
**CLP**	12.07**	20.45**	8.21**	377.91**	0.82	14.08**		40.03**	10.62**	298.68**	3.99**
**PPC**	0.71	0.44	0.64*	8.09**	0.13	0.79		0.77	0.78*	2.02*	0.37
**PPP**	96.88**	42.6**	63.44**	3516.6**	2.35	101.81**		36.89**	67.06**	3602.8**	3.17
**PH (cm)**	147.87**	338**	143**	249.54**	2.46	170.69**		388.25**	164.35**	350.62**	5.99
**DM**	100.09**	301.86**	96.98**	4.72	2.97	102.33**		306.33**	99.16**	1.83	16.23
**SYPP (g)**	29.96**	31.77**	30.09**	13.11**	0.76	29.69**		27.9**	29.76**	26.79**	2.55
**SW (g)**	9.09**	176.01**	5.8**	0.84	0.6	8.93**		171.52**	5.64**	2.32	1.09
**SPP**	2.17**	0.86	2.2**	2.6**	0.21	2.35**		1.55*	2.35**	3.35*	0.78

*p < 0.05

**p < 0.01

DF: Degrees of freedom, DFF: Days to 50% flowering, PB: Primary branches, CLP: Number of clusters per plant, PPC: Number of pods per cluster, PPP: Number of pods per plant, PH: Plant height, DM: Days to 80% of maturity, SYPP: Seed yield per plant, SW: Seed weight, SPP: Number of seeds per pod

Descriptive statistics of 10 quantitative traits indicated high significant variation was present among all the accessions (Table 2 and S3 Table). Accessions EC340283 and IC341944 flowered in 53 days and were identified as early flowering accessions whereas, accessions EC59489 (87 days) and IC341951 (88 days) were late flowering. The mean value of best performing check (Totru Local) was 53 days. Five accessions flowered earlier than the check. The number of primary branches (PB) among all the traits showed a high coefficient of variations (19.2%). In both the year accessions IC89957 and EC340283 possess the lowest number of primary branches (1). Accessions IC341954 have the highest number of primary branches (4), followed by IC341945 (3.75) while in Totru Local three PB were recorded. Fifteen accessions had more than the check and 46 accessions were more than the mean value of two PB. The number of clusters per plant (CLP) varied significantly among the accessions. Accession EC000264 possess the highest CLP (18), followed by IC341939 (17), while the lowest CLP was recorded in EC340283 (4.67) followed by IC341946 (4.65). The best-performing check Grams Local 2 had 15.29 CLP. Five accessions were above this check and 39 accessions exceeded the average 8.64 CLP, which is significant. Significant variability was seen in number of pods per cluster (PPC) across the accessions. Accession IC140846 (5.9) had highest PPC whereas, EC340257 and IC469171 have lowest (1.41). The best performing check Grams Local 2 had 3.17 PPC. There was highly significant variability identified for the number of pods per plant (PPP) across the accessions. Among all, accession IC469175 (41.9) showed the highest PPP followed by EC34264 (41.15) and EC000264 (37.67). However, genotype EC30256 (7.9) had the lowest followed by IC341946 (8.4). The best performing check Grams Local 2 was having 34.4 PPP. 7 accessions were better than the checks and 39 accessions were more than the average value 18.36 PPP.

**Table 2:** Descriptive statistics of quantitative traits of adzuki bean for both season (2020 & 2021).

Traits	Location	Mean	Range	SE	SD	CV
DFF	Season 1	59.31	52.95–88.62	0.58	5.87	2.46
	Season 2	60.15	51.76–89.67	0.56	5.68	1.83
PB	Season 1	2.14	0.91–4.30	0.07	0.71	19.29
	Season 2	2.17	0.85–3.86	0.07	0.70	19.12
CLP	Season 1	8.25	3.75–18.72	0.29	2.93	16.08
	Season 2	9.02	3.16–19.05	0.33	3.37	7.00
PPC	Season 1	2.54	1.29–5.76	0.08	0.80	11.75
	Season 2	2.56	0.92–6.26	0.09	0.92	20.69
PPP	Season 1	18.42	7.96–41.46	0.80	8.10	5.73
	Season 2	18.31	6.75–42.50	0.82	8.34	8.68
PH (cm)	Season 1	54.28	26.90–94.33	1.20	12.20	2.47
	Season 2	53.54	29.59–97.14	1.21	12.27	6.33
DM	Season 1	110.96	91.10–130.43	0.99	10.06	1.68
	Season 2	110.18	85.75–129.52	0.98	9.99	4.11
SYPP (g)	Season 1	12.59	5.12–45.38	0.53	5.39	9.14
	Season 2	12.99	4.85–48.98	0.54	5.46	13.05
SW (g)	Season 1	11.11	4.42–20.92	0.25	2.57	6.21
	Season 2	11.05	5.83–19.08	0.25	2.49	8.00
SPP	Season 1	7.61	4.40–10.48	0.14	1.44	8.71
	Season 2	7.68	4.35–10.92	0.16	1.58	7.55

DFF: Days to 50% flowering, PB: Primary branches, CLP: Number of clusters per plant, PPC: Number of pods per cluster, PPP: Number of pods per plant, PH: Plant height, DM: Days to 80% of maturity, SYPP: Seed yield per plant, SW: Seed weight, SPP: Number of seeds per pod.

Accessions IC469174 (95.4 cm) was recorded with the highest plant height (PH) and EC340283 (28.99 cm) observed the lowest PH. The shortest check Totru Local, recorded a PH of 51.77 cm. Forty-seven accessions were shorter than this check and 54 accessions were shorter than the mean value of 53.91 cm. Accession EC340259 matured very late (128 days) followed by SMLAB8 (126). Accession, EC120460 matured very early (90 days) followed by IC341941 (91 days). Check HPU-51 matured in 103 days, 27 accessions matured earlier than the check and 51 were earlier than the average of 110.57 days. For seed yield per plant (SYPP), EC15256 recorded the highest SYPP (47.17 g) while IC469173 recorded the lowest SYPP (6.36 g) and check HPU-51 yielded 13.39 g. Thirty-seven accessions yielded more than the check i.e. HPU-51 and 42 accessions yielded above average yield 12.79 g. For 100-seed weight (SW), SMLAB7 recorded a high SW (19.42 g) and IC251353 had a low SW (5.33 g). SW of Check Grams Local 2 was 15.37 g, six accessions were more than the check. The maximum number of seeds per pod (SPP) was recorded for IC251353 (10) while the minimum was in EC340244 (5.48). Check Totru Local had 7.49 SPP, 57 accessions had more SPP than the check and 43 accessions were more than the average SPP 7.64.

Table 3 presents important genetic parameters such as PCV, GCV, H^2^ and GAM for all the traits. Among the quantitative traits, DFF and DM showed low PCV and GCV. SW and SPP exhibited medium PCV and GCV and remaining traits PB, CLP, PPC, PPP, PH and SYPP demonstrated high PCV and GCV values. High H^2^ values across all traits were observed, indicating a significant genetic influence on their variation. GAM was medium for DFF and DM whereas, it was high for the remaining eight traits.

**Table 3:** Genetic variability analysis for 10 quantitative traits of adzuki bean for both the season 1 (2020) and 2 (2021).

Trait	Mean	PCV (%)	State	GCV (%)	State	H^2^ (%)	State	GAM (%)	State
DFF	59.73	9.71	Low	9.61	Low	97.98	High	19.62	Medium
PB	2.15	30.13	High	25.98	High	74.36	High	46.22	High
CLP	8.64	34.17	High	33.12	High	93.91	High	66.2	High
PPC	2.55	30.56	High	28.73	High	88.36	High	55.72	High
PPP	18.36	42.87	High	42.76	High	99.48	High	87.99	High
PH (cm)	53.91	22.69	High	22.63	High	99.49	High	46.57	High
DM	110.57	8.84	Low	8.77	Low	98.22	High	17.92	Medium
SYPP (g)	12.79	41.11	High	40.78	High	98.39	High	83.45	High
SW (g)	11.08	19.68	Medium	19.12	Medium	94.43	High	38.33	High
SPP	7.64	17.97	Medium	17.17	Medium	91.25	High	33.83	High

PCV: Phenotypic coefficient of variation, GCV: genotypic coefficient of variation, H^2^ heritability in a broad sense (%), GAM: genetic advance percent of means.

Based on two years evaluation, promising accessions were selected for the traits analysed (Table 4). The accessions with early flowering (DFF- 54 days) were EC340283; IC341944; EC340257; EC18256. Accession EC15256 (47.2 g), EC000377 (23.5 g) and EC120460 (20.9 g) were found promising for seed yield per plant. Accessions IC469175 (41.9), EC34264 (41.1) and EC000264 (37.6) recorded highest PPP. Accession EC340283 was found to be promising for multiple traits like DFF (53.5 days), PH (28.99 cm) and DM (93 days).

**Table 4:** List of promising accessions of adzuki bean with desirable traits based on morphological characterization.

Traits	Accessions
Days to 50% flowering (DFF)	EC340283; IC341944; EC340257; EC18256 (53.5 days)
Number of primary branches (PB)	IC341954; IC341945; IC469175 (4),
Number of clusters per plant (CLP)	EC000264 (18.4), IC341939 (17.2) and IC16761 (16.91)
Number of pods per cluster (PPC)	IC140846 (5.9), IC341942 (3.9) and EC36973A (3.9)
Number of pods per plant (PPP)	IC469175 (41.9), EC34264 (41.1) and EC000264 (37.6)
Plant height (cm) (PH)	EC87896 (28.30 cm), EC340283 (28.99 cm) and IC341941 (32.81 cm)
Days to 80% maturity (DM)	EC120460 (90 days), IC341941 (91 days) and EC340283 (93 days)
Seed yield per plant (g) (SYPP)	EC15256 (47.2 g), EC000377 (23.5 g) and EC120460 (20.9 g)
100-seed weight (SW)	SMLAB7 (19.42 g), EC000248 (17.56 g) and EC340271 (16.40 g)
Number of seeds per pod (SPP)	EC340240 (10), IC455396 (9.9) and IC341955 (9.9)

An accession EC340283 was found to be unique and found to be promising for multiple traits such as DFF, PH and DM. Genotype IC469175 was identified as promising for important yield traits PB and PPP. A unique accession EC120460 was found to be promising for yield and plant architecture trait DM and SYPP.

Accession EC340283 stands out for its exceptional performance across multiple traits including days to first flowering (DFF), plant height (PH), and days to 80% maturity (DM) production. This genotype shows potential for robust growth and productivity, making it a valuable candidate for breeding programs focused on enhancing crop resilience and yield. Genotype IC469175 demonstrates promise for pivotal yield-related traits such as the number of primary branches (PB) and pods per plant (PPP), indicating its suitability for increasing overall yield potential. Another noteworthy accession is EC120460, known for its high seed yield per plant (SYPP) and early maturity (DM), which are critical factors for optimizing harvest efficiency and ensuring timely crop cycles. Highlighting these unique accessions underscores their importance in advancing adzuki bean breeding efforts aimed at addressing global food security challenges through improved crop performance and resilience.

### Principal component analysis (PCA)

PCA was carried out for all 10 quantitative traits for two seasons of mean data. Loadings were calculated to detect the underlying link between the data structure after representative principal components (PCs) were recognized based on sample grouping and variability to each trait was calculated (Table 5). An Eigenvalue was assigned to each PC that accounts for a fraction of the variation in the dataset. Each Eigenvalue has an eigenvector linked to it, which defines the variation within the main components. A Scree plot was used to plot the eigenvalues of factors or principal components. The scree plot containing the principal components based on eigenvalues (>1) is shown in S2 Fig. In our study, 66.2% of the overall variation was accounted by the first four principal components (PC1, PC2, PC3 and PC4; eigenvalues >1; S2 Fig). The PC1 contributed 28.1% for major variability exhibited by all the accessions, followed by PC2 which contributed 15.4%, PC3 12.1% and PC4 10.6%.

**Table 5:** Contributions of variables for each principal component of PCA.

Traits	PC1	PC2	PC3	PC4
DFF	0.563	1.785	4.289	22.850
PB	16.06	3.105	8.179	0.008
CLP	23.62	2.050	0.161	0.429
PPC	13.55	1.556	0.167	1.856
PPP	30.44	4.251	0.068	0.004
PH	11.98	1.212	0.472	0.169

**Fig 1:**
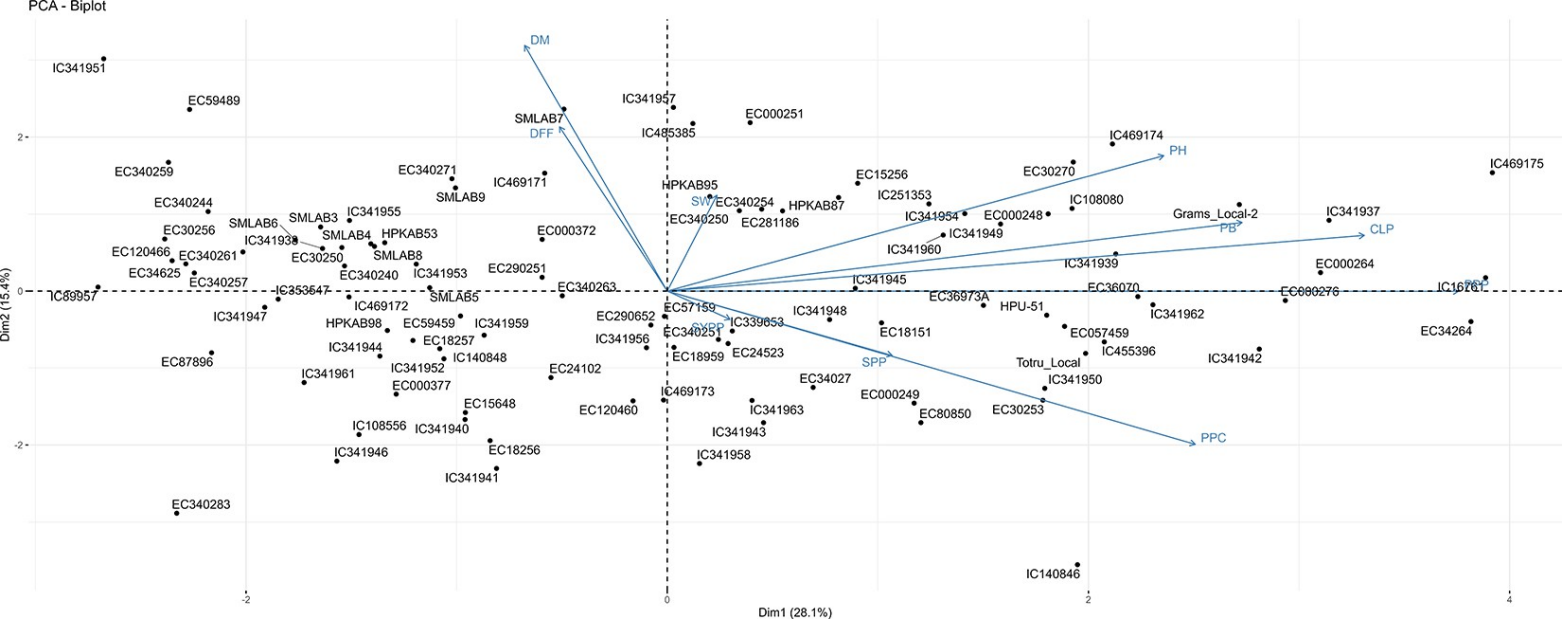
PCA biplot indicating the distribution of 10 quantitative traits in 100 accessions of adzuki bean based on their calculated component loading values.

In Principal Component 1, the traits PPP (30.44), CLP (23.62), PB (16.06) and PPC (13.55) have showed maximum values which indicate that PPP, CLP, PB, PPC traits had large contributions in variability. The traits i.e. PPP, CLP, PB, PPC are beneficial and can be utilized in further breeding program. According to principal component analysis the traits PH (11.98) and DFF (0.563) are underutilized. In the principal component biplot, the angle between the two vectors was lower signifying the positive association among the traits (Fig 1). The probability of a correlation declines as the vectors make a 90-degree angle, and the correlation becomes negative when they make a 180-degree angle. As a result, accessions could be categorized according to the relationship between variables and the distinctiveness of dominant characteristics.

### Correlation analysis

**Fig 2:**
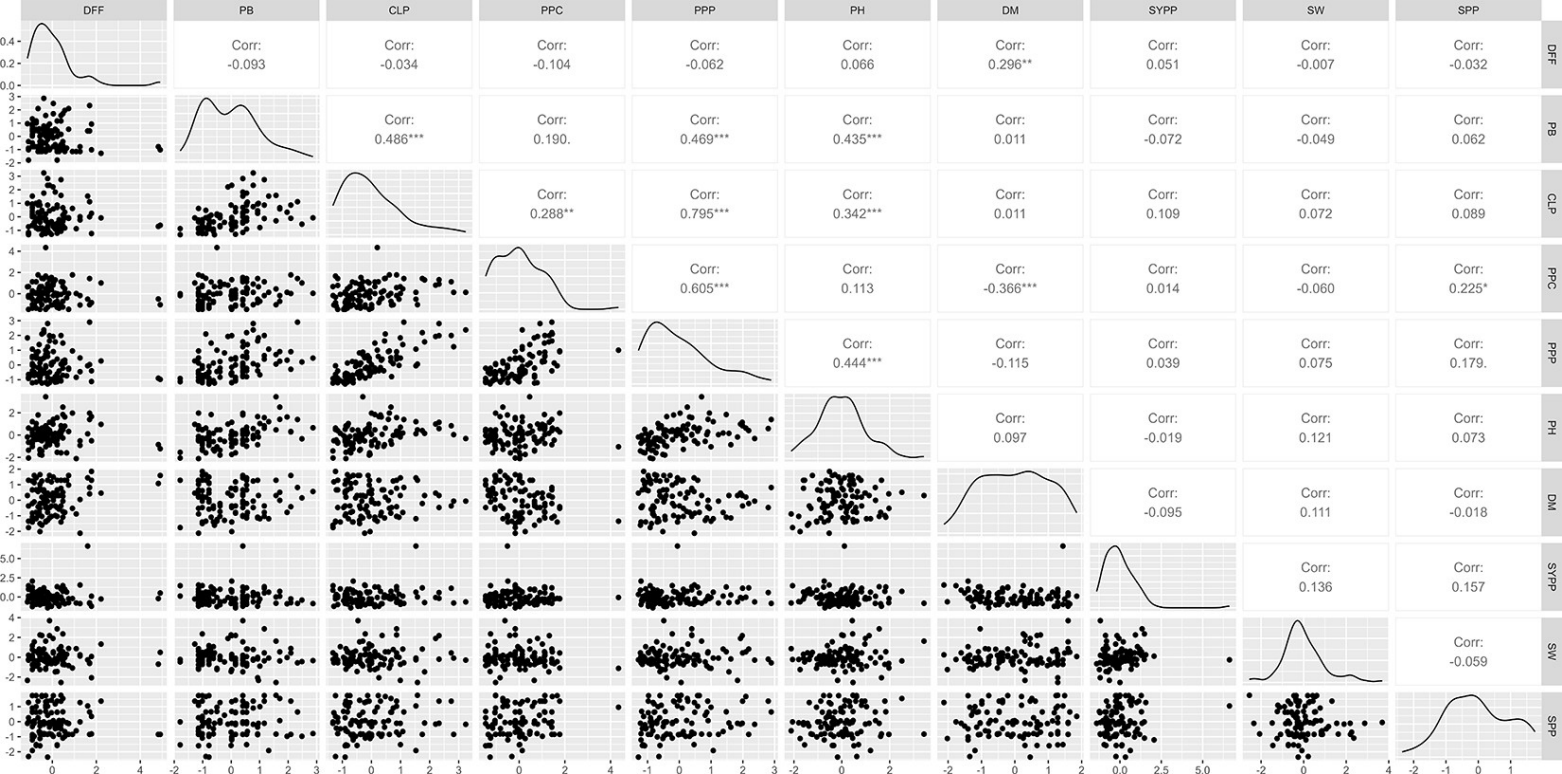
Correlation matrix of quantitative traits where; DFF: Days to 50% flowering, PB: Primary branches, CLP: Number of clusters per plant, PPC: Number of pods per cluster, PPP: Number of pods per plant, PH: Plant height, DM: Days to 80% of maturity, SYPP: Seed yield per plant, SW: Seed weight, SPP: Number of seeds per pod. Ns = non-significant, *p < 0.05, **p < 0.01, ***p< 0.001.

The correlation matrix of 10 quantitative trait variables was computed using the Karl Pearson correlation coefficient. The correlation coefficient matrix of quantitative traits presented in Fig 2. CLP was high positive significantly correlated with PB (r = 0.486**) and significantly correlated with PPC (r = 0.288*). Pods per plant were positive significantly correlated with PB (r = 0.469**), CLP (r = 0.795**) and PPC (r = 0.605**). PH showed a positive correlation with PB (r = 0.435**), CLP (r = 0.342**), and PPP (r = 0.444**). DFF was positive significantly correlated with DM (r = 0.296*) and negative significantly correlated with PPC (r = -0.366**). CLP exhibited positive correlations with DM, SYPP, SW, and SPP, although these correlations were not statistically significant. While DFF exhibited negative and non-significant correlations with the traits PB, CLP, PPC, and PPP.

#### Hierarchical cluster analysis (HCA) of quantitative traits

**Fig 3:**
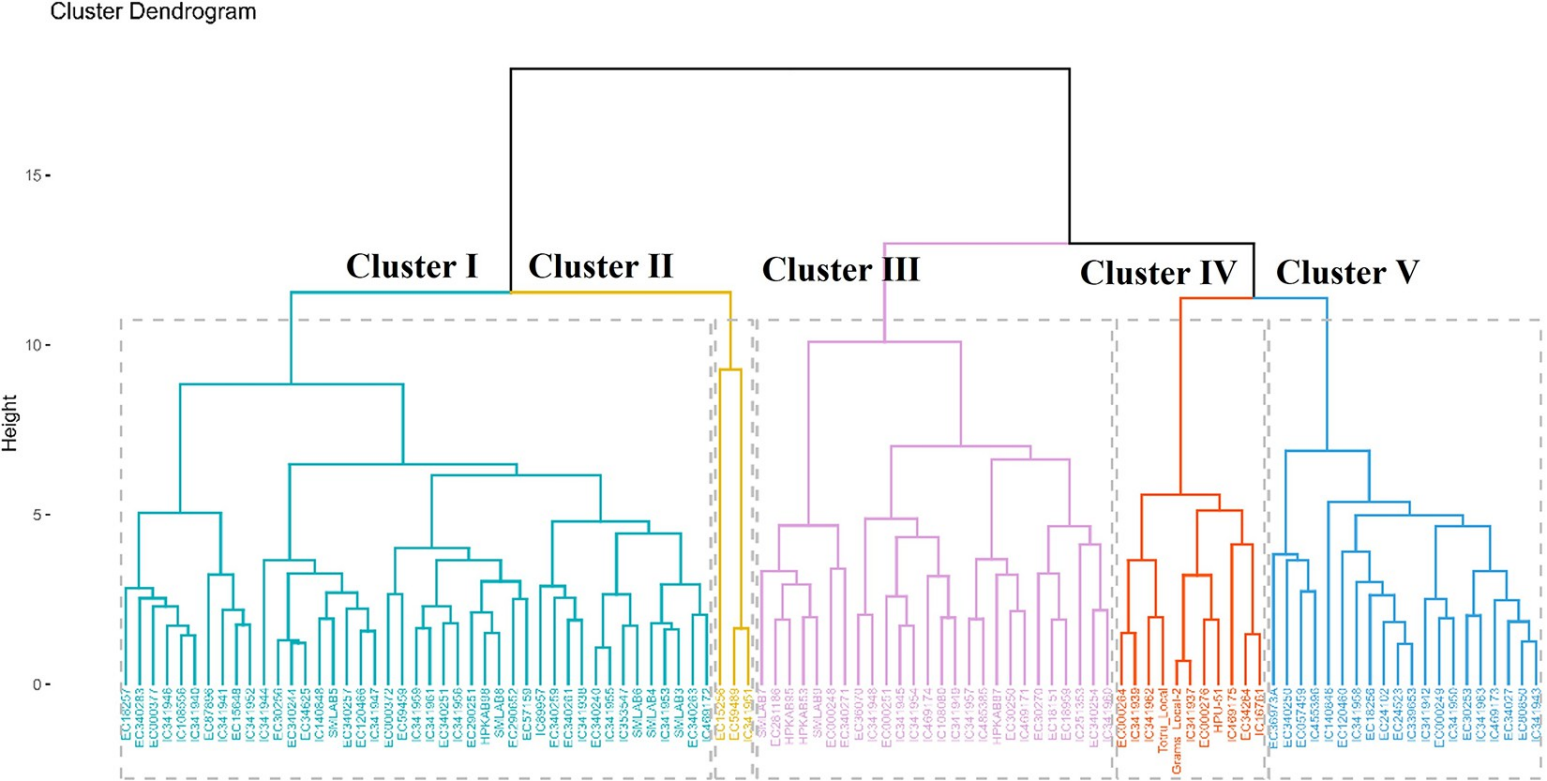
“k- means clustering” of 100 accessions and three checks of adzuki bean based on ten quantitative traits.

The cluster analysis based on 10 quantitative traits divided all the 100 accessions of adzuki bean into two major clusters. At the distance of 10, all accessions were grouped into five clusters viz., cluster I to cluster Ⅴ (Fig 3). For each cluster, the respective grouped accessions mean values were calculated for all the quantitative traits and presented in Table 6.

**Table 6:** The number of accessions and average values of five clusters for ten different quantitative traits for 100 accessions of adzuki bean.

Clusters	Count	DFF	PB	CLP	PPC	PPP	PH	DM	SYPP	SW	SPP
1	43	58.85	1.72	6.62	2.24	12.27	46.91	110.83	12.29	10.83	7.33
2	3	81.50	1.86	8.83	2.02	13.13	45.92	123.83	24.81	10.73	7.36
3	26	59.46	2.74	9.51	2.19	18.99	63.43	114.50	11.84	11.85	7.25
4	11	59.31	2.61	14.79	3.23	34.76	59.63	110.76	12.42	11.33	7.69
5	20	64.78	2.23	9.94	2.42	19.79	53.97	114.98	15.34	11.18	7.41

Among all the clusters, cluster Ⅰ was the largest cluster comprising 43 accessions and distinguished by early flowering accessions (59 days), lower PB (1.72), lowest CLP (6.62), lowest PPP with other traits in moderate values. Cluster Ⅱ was the smallest with three accessions and characterized by late flowering (81.50 days), short PH (45.92 cm), late maturing (124 days), and highest SYPP (24.81 g), with the lowest SW trait (10.73 g) accessions. Cluster Ⅲ with 26 accessions, categorized by highest values for PB (2.74), PH (63.43 cm) and SW (11.85 g) and lowest values for SPP (7.25). Other traits in this cluster showed moderately high performance. Cluster Ⅳ contained 11 accessions which were characterized by a maximum number for CLP (14.79), PB (3.23), PPP (34.76), SPP (7.69) and early maturing (110.76 days) and remaining traits were in moderate quantity. Cluster V, comprising 20 accessions, was characterized by moderate amounts across all ten traits. Overall, these cluster analyses provide valuable insights into the diversity of adzuki bean accessions based on their quantitative traits.

#### Variation for qualitative traits in 100 accessions of adzuki bean

**Fig 4:**
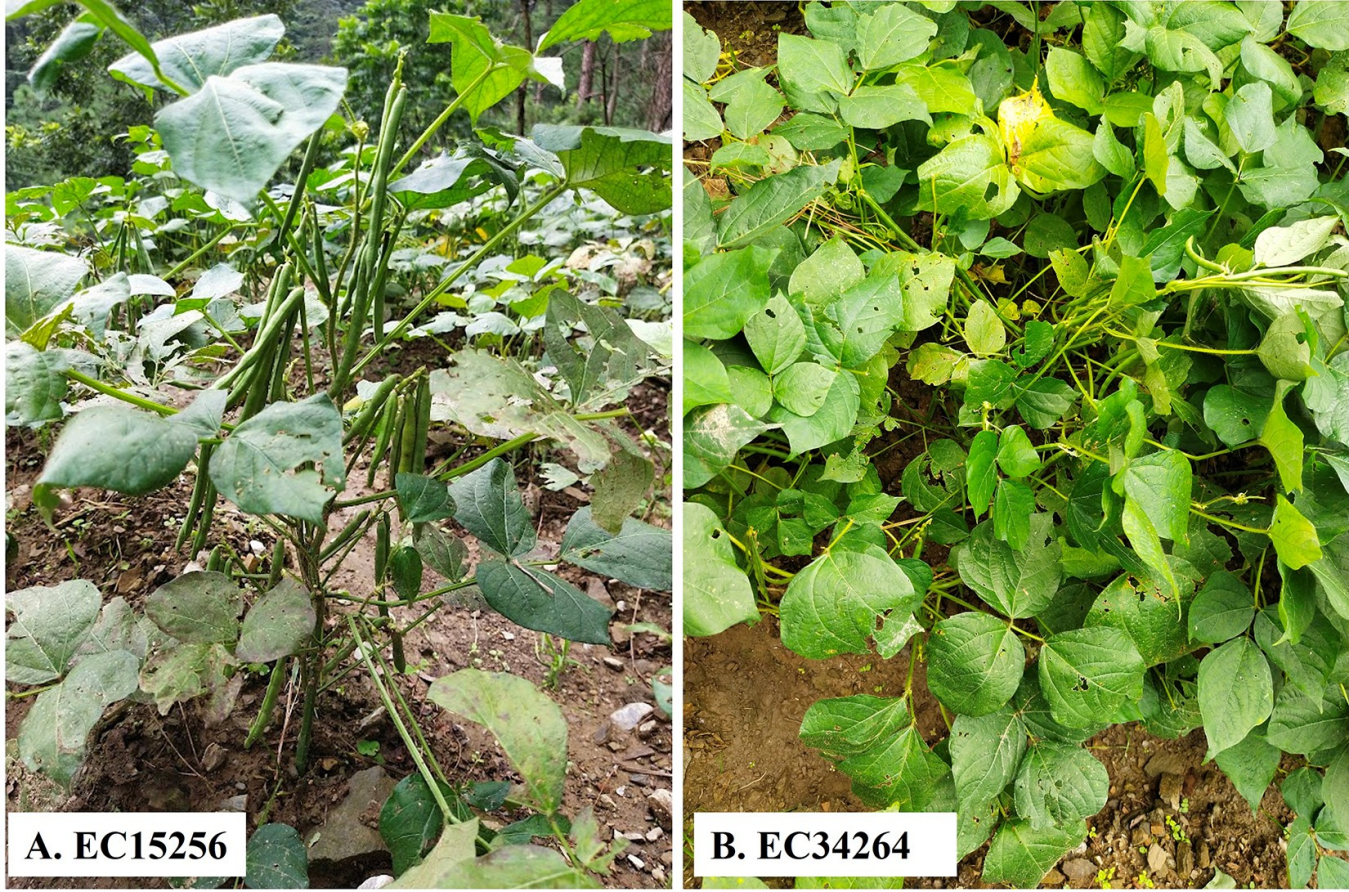
A&B: Representing variations in plant growth habits observed in adzuki bean accessions A- erect growth habit (EC15256); B- semi-spreading growth habit (EC34264).

**Fig 5:**
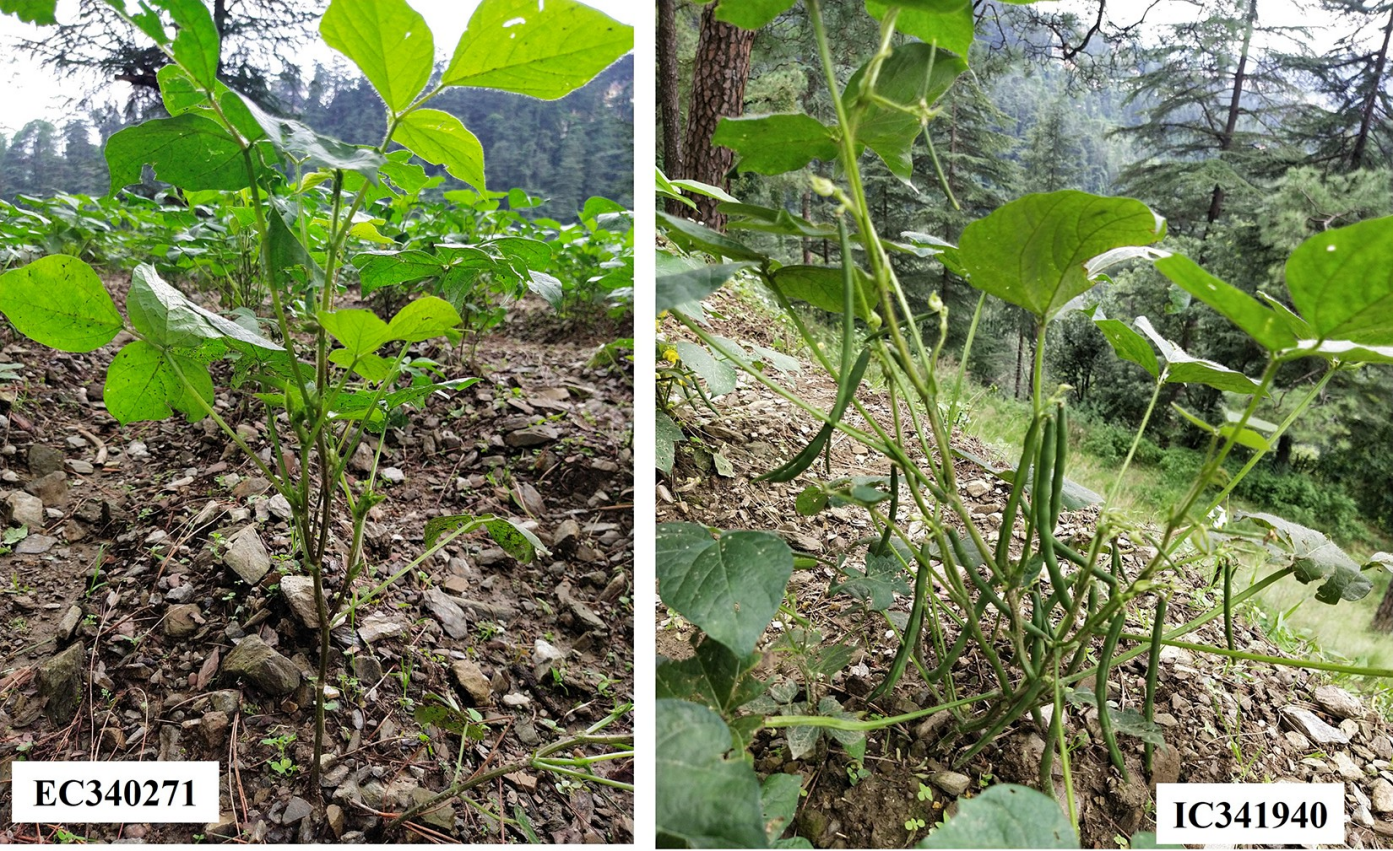
Representing variations in stem colour of adzuki bean. A- purple coloured stem (EC340271); B- Green coloured stem (IC360528).

**Fig 6:**
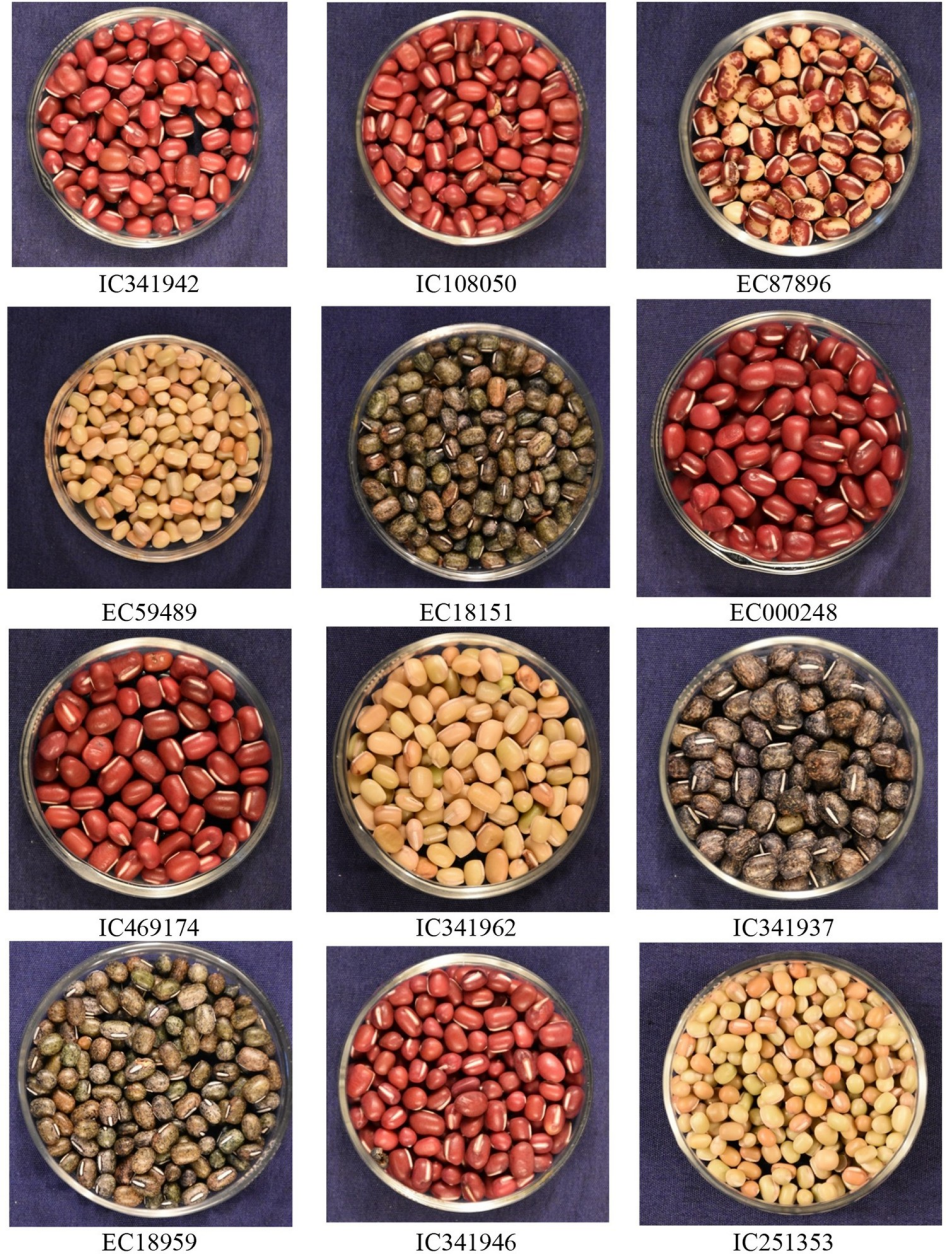
Figure representing diversity in seed coat colour in adzuki bean molecular characterization using SSR.

Data for 12 qualitative traits were recorded, among them only five traits *viz*., early plant vigour, plant growth habit, leaf colour, stem colour and seed coat colour showed variation within the species. However, the remaining qualitative traits, including leaf surface, leaflet shape, stem surface, flower colour, pod angle, and pod surface, did not exhibit significant variability within the studied species (S4 Table, Figs 4–6 and S3 Fig).

For early plant vigour, the majority of the accessions (77) showed good vigour, 13 accessions were very good and 10 recorded poor vigour. For plant growth habit trait, 9 accessions were semi-spreading and remaining with erect height. Three types of leaf colours were observed, 4 were yellowish green, 47 were dark green and 49 accessions reported green coloured leaves. The majority of accessions (97) showed green stem colour and only 3 were purple pigmented. Out of 100 accessions, 87 accessions were with red seed coat colour, 8 with green and black mottled and 4 yellow coloured and only 1 accession showed half red and half yellow mottled seeds.

Genetic diversity analysis of adzuki bean with 22 polymorphic SSR markers revealed significant variations among the accessions (Table 7 and S4 Fig). A total of 50 scorable amplified fragments with an average of 2.27 bands per primer ranging between 80 to 250 bp were generated by 22 SSR makers. The amplification of alleles per SSR primer was observed at a maximum of 3 alleles and a minimum of 2 alleles. The gene diversity varied from a maximum of 0.569 (AB128100) to a minimum of 0.030 (VR016) with a mean value of 0.186. The observed heterozygosity ranged from 0.00 (E722-432) to 0.989 (AB128100) with an average of 0.134. Major allele frequency (MAF) ranged between 0.495 to 0.985, where primer VR016 showed maximum MAF and AB128100 showed minimum. Polymorphic information content (PIC) ranged from 0.03 (VR016) to 0.46 (AB128100) with an average of 0.16.

**Table 7:** List of SSR primers used for genotyping of adzuki bean and their allelic diversity.

No.	Marker	Primers Sequence	AT	AN	MAF	GD	H	PIC
**1**	E7233	F-TTGATTACCAAAACACTGCTTCA R-TTATGTCGACCTTATCCAAATCG	51	3	0.776	0.370	0.188	0.336
**2**	E722-432	F- GCAGTTCTTCTCGTTCTTCTCTG R- AAAGTCCTCTAAAGCACAATCCC	51	2	0.925	0.139	0.000	0.130
**3**	E6237-2652	F- TTTCAGCATCATTTATTTCCAGT R- TGTCTCGAAATCATAGACACAGTT	51	2	0.976	0.047	0.000	0.046
**4**	E5350	F-AAACAAAGCAGAAGAGCAACAAC R- GGGAGATAGAGTTGAGGGAGAAG	51	2	0.954	0.088	0.092	0.084
**5**	E5057-1800	F-AGTTCCGAAATTGGGTATTTGTT R- ACACCCACAATAACTATTCGCAG	51	2	0.962	0.072	0.075	0.070
**6**	E4940	F- TTGATTACCAAAACACTGCTTCA R- TTATGTCGACCTTATCCAAATCG	51	2	0.616	0.473	0.012	0.361
**7**	E4852	F-GCAGTTCTTCTCGTTCTTCTCTG R- AAAGTCCTCTAAAGCACAATCCC	51	2	0.955	0.086	0.090	0.082
**8**	E4794	F- TCTGATACAAAGAAAATCAGGCG R- CAAGAAACCAGACGTGATACTCC	51	2	0.967	0.063	0.065	0.061
**9**	E1961-971	F- GACGTTCACTTGTTCATGTACCC R- ACTATGCGTTCTGAAACCAAAAA	51	2	0.940	0.113	0.000	0.106
**10**	E18280	F- CACAAATTCTCAAACTCAAACCC R- GAGACACTCCTTGTAGGAAACCA	51	2	0.919	0.150	0.047	0.138
**11**	E157-81	F- CCAGGGAAAATGAAAATGAAAA R- CTCGCTCATTATCATCTCCGAC	51	3	0.913	0.163	0.081	0.156
**12**	E1248-711	F-GCAGTTCTTCTCGTTCTTCTCTG R- AAAGTCCTCTAAAGCACAATCCC	51	2	0.967	0.063	0.065	0.061
**13**	G314	F- CAGCGATCGAGGTTCCTAAG R- AAAGGACGGGTGTGTGACTC	51	3	0.889	0.199	0.051	0.181
**14**	AB128093	F-CCCGATGAACGCTAATGCTG R-CGCCAAAGAAACGCAGAAC	48.6	3	0.818	0.305	0.031	0.269
**15**	VR400	F-ATCATAGATAGGGGACCAACCC R-ATCTTAGGGAGTCTTCGAGGGA	55	2	0.960	0.078	0.000	0.075
**16**	VR016	F-AGGAGAAATTGTTGTTGTTCGG R-GTGTTGATTGTTAGGGAGGGAG	49.1	2	0.985	0.030	0.030	0.029
**17**	VR413	F-GAGAAACCTTGGAGTTGGAGG R-GCCTGTCAAGAAGGAACCTAAA	55	2	0.960	0.077	0.000	0.074
**18**	VR108	F-GCTCCAACACTCACTCACAAAC R-CAGAAATGCAGGAAAAGAGAGG	52	2	0.985	0.030	0.030	0.029
**19**	VR032	F-ATATCAGCCATTGTTGCTTTCC R- TTCCCAGTTCAGACAACCAAGT	54	2	0.970	0.058	0.020	0.057
**20**	VR140	F-GGTGTTGTTGTTGAGGAATGAA R-AACATTGAGGACCCACATATCC	52	2	0.510	0.500	0.980	0.375
**21**	AB-128079	F-AGCGAGTTTCGTTTCAAG R-GCCCATATTTTTACGCCCAC	48	3	0.719	0.415	0.104	0.345
**22**	AB128100	F-CATCTTCCTCACCTGCATTC R-TTTGGTGAAGATGACAGCCC	48.6	3	0.495	0.569	0.990	0.476
	Mean			2.27	0.871	0.186	0.134	0.161

(F-forward, R-reverse, AT-annealing temperature (˚C), AN-allele number, MAF-major allele frequency, GD-gene diversity, H-heterozygosity, PIC-Polymorphic information content)

#### Hierarchical cluster analysis of adzuki bean using SSR markers

**Fig 7:**
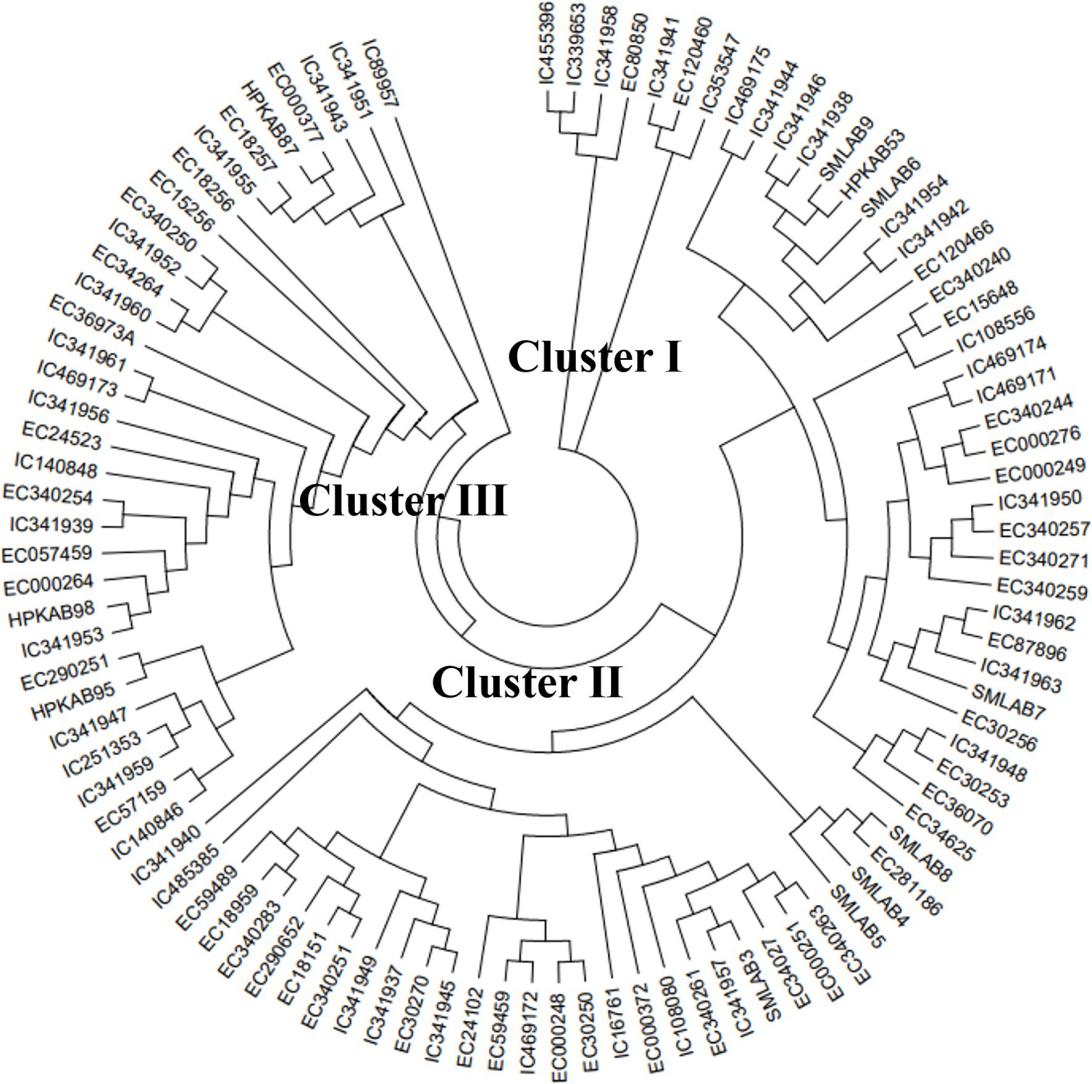
Dendrogram based on Nei genetic diversity using neighbor joining tree method of clustering, depicting genetic diversity and relationship between 100 accessions of adzuki bean as revealed by polymorphism of 22 SSRs.

The DNA polymorphism revealed by 22 primers was used for studying the clustering pattern of adzuki bean accessions. All the 100 accessions were grouped into three major clusters viz., cluster I, cluster II and cluster Ⅲ (Fig 7). Cluster I is the smallest cluster and was composed of two subclusters, one of which had four and the other has three accessions, having a total of seven accessions. Cluster II again subclustered into three clusters, subcluster one has 31 accessions, subcluster 2 has only four accessions and subcluster 3 has 26 accessions. Cluster Ⅲ again subclustered as two clusters, subcluster Ⅰ have only one genotype and subcluster 2 had 31 accessions.

#### Population structure analysis

**Fig 8:**
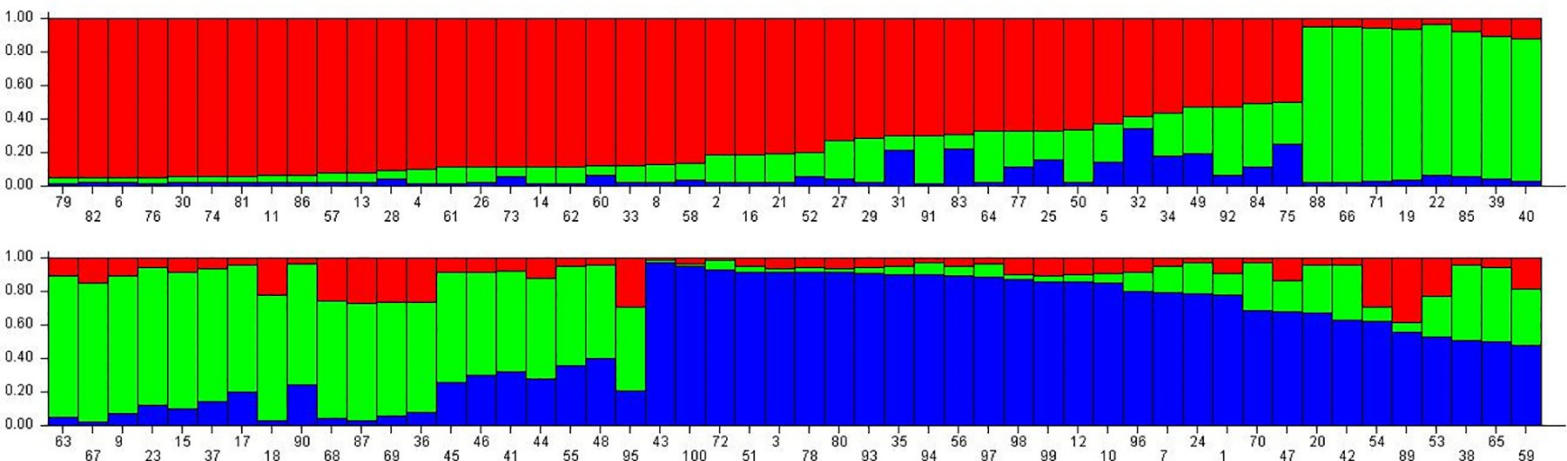
Model-based clustering of 100 accessions of adzuki bean detected based on 22 SSR markers. An accession is represented by each bar. The various colour bars (red, green and blue respectively) reflect various genetic groups, while the combination of colours in a bar indicates admixture.

The 100 accessions of adzuki bean were assembled into three population based on the (delta) LnPD values plotted against the corresponding K values to determine the ideal K (S5 Fig), which is consistent with the results as shown in the dendrogram (Fig 5). The 100 accessions were distributed into three populations. Population Ⅰ consists of 16 admixture and 26 pure individuals; in population Ⅱ had 13 admixture and 15 pure; population Ⅲ had 10 admixture and 20 pure individuals (S5 Table and Fig 8).

#### Analysis of molecular variance (AMOVA)

AMOVA of 100 accessions of adzuki bean was executed to analyze the genetic diversity distribution within and between the populations of adzuki bean. The analysis revealed maximum diversity within individuals was 48%, diversity among individuals was 40% and diversity among populations was 12%. Variation within individuals was highest (1.419), among individuals (1.189) and lowest among populations (0.364) (Table 8).

**Table 8:** Analysis of molecular variance (AMOVA) of 100 accessions of adzuki bean.

Source	Df	SS	MS	Est. Var.	%
Among Populations	2	54.75	27.37	0.364	12
Among Individuals	96	364.3	3.796	1.189	40
Within Individuals	99	140.5	1.419	1.419	48
Total	197	559.7		2.972	100

Df: Degrees of freedom, SS: sum of squares, MS: mean square, Est. Var.: estimated variance

#### Principal Coordinate Analysis (PCoA)

**Fig 9:**
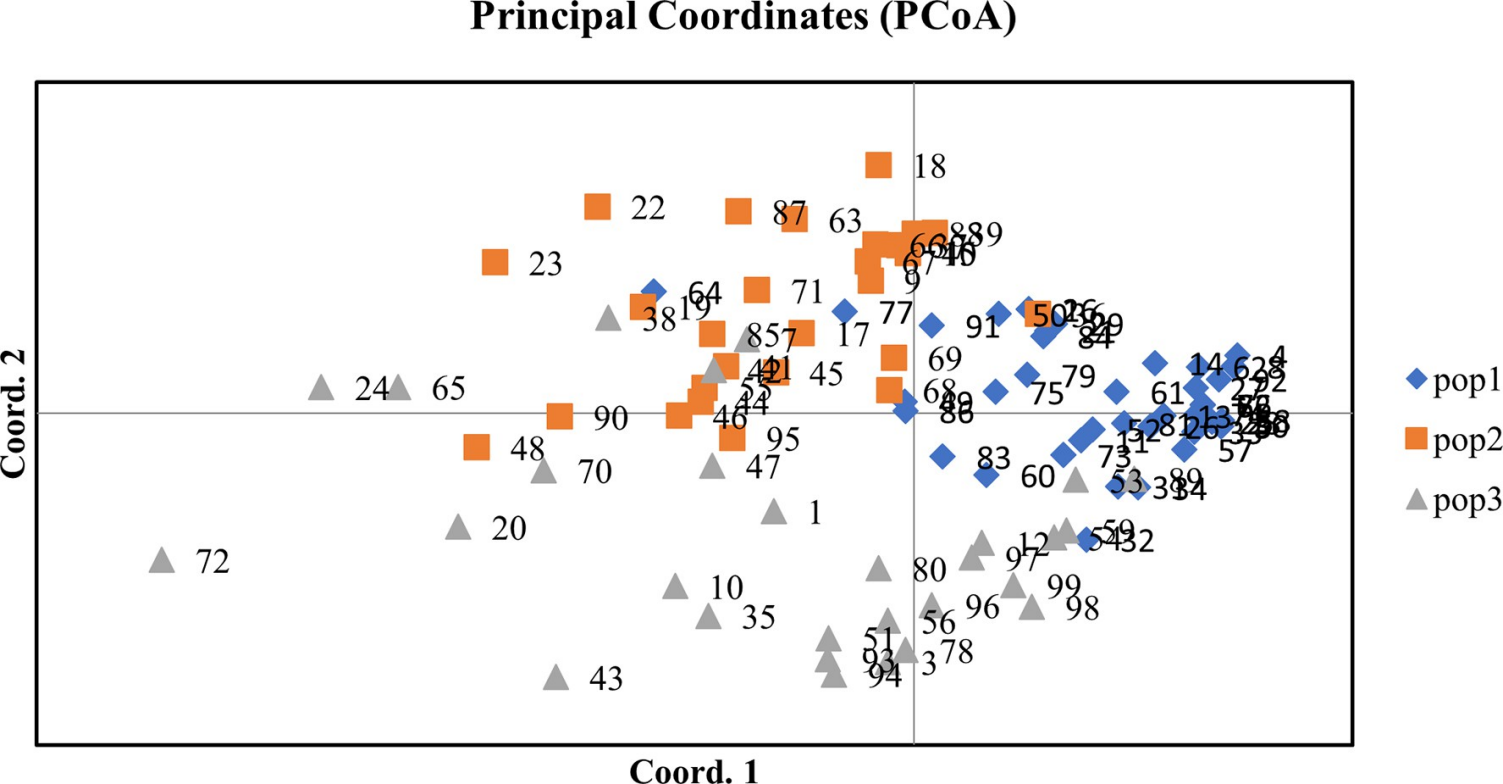
Principal Coordinate Analysis (PCoA) of 100 accessions of *Vigna* angularis.

To describe the genetic variance using multidimensional association, PCoA was carried out. The first three PCoA axes explained 11.59%, 9.43%, and 8.38% of the variation, respectively and the cumulative percent variation was 29.4%. The PCoA also grouped 100 accessions of adzuki bean into three clusters, which compliments the results of cluster analysis and population structure analysis. The results of PCoA disclosed that high genetic variability existed in adzuki bean accessions (S6 Table and Fig 9).

## Discussion

Adzuki bean is one of the important underutilized grain legumes crops nevertheless its potential is not fully explored because of the lack of recognition of its health benefits among the people and the lack of improved cultivars for its production and area expansion [25]. The inclusion of adzuki bean in food baskets increases the diversification of diet and adds supplementary nutrients to humans [26]. In the era of climate change and global pandemics, adzuki bean serves as a sustainable, resilient, environment-friendly crop that acts as nutraceutical food besides having export potential.

Agro-morphological evaluation of the plant genetic resources is the first step of crop varieties development. The generated data also helps in grouping the accessions which helps to develop reference set or core sets and helps to decide the genebank to supply the germplasm more suited to the breeders or other researchers’ needs, and identification of redundancy and gaps in the collections. Many workers previously have done morphological diversity assessments of adzuki bean [4, 27–33]. All these works emphasized exploitable variations that exist in the adzuki bean which is the basic requirement of crop improvement. Screening has been done to find the resistant sources for biotic and abiotic stress viz., for salt tolerance [31, 34], and waterlogging tolerance [35]. Adzuki bean is a highly photosensitive crop and requires short days for flowering, play a key role in its latitudinal adaptations. Efforts should be made to screen photo-insensitive germplasm to develop new varieties and expand its cultivation area [36, 37]. Researchers revealed that the nutritional profile of adzuki bean is equivalent to major pulses and very close to mungbean and cowpea [4, 26, 32, 38–40]. Anti-nutritional factors such as phenols and phytic acid content are lower in adzuki bean compared to cowpea and soybean [41, 42]. Specifically, phytic acid was found to be lower in adzuki bean milk compared to soybean milk [42].

In the present study, quantitative traits related to plant architecture such as PH showed significant variations ranging between 28.4 cm to 95.7 cm with an average of 53.9 cm. In the previous studies, PH ranged between of 13.5 to 297 cm [32, 43]. PB in the current study ranged from 1 to 4, contrasting with the results reported by Hu et al., (2022) [43], where a PB range of 0 to 10 with a mean of 2.4, due to the inclusion both CWR and landraces of adzuki bean. PH and PB play a significant role, it influences DFF and DM, CLP, PPP and SYPP [27, 43]. In our study DFF showed wide variations between 52.3 to 89.7 days and DM, 90 to 128.5 days and an average of 110.6 days. Earlier workers also reported wide variations in the DFF and DM and reports were consistent with our work. A 47-to-77-day range for flowering and 87 to 127 days for maturity in 252 germplasm lines was reported by Desta et al., (2023) [33]. Hu et al., (2022) [43] reported 33.7 to 86.3 days for flowering and 63.3 to 106.0 days for maturity in 27 wild relatives and 448 landraces. Similarly, considerably broader values for DFF 51 to 110 days and DM 81 to 150 days were recorded earlier [28]. The diversity in DFF and DM was primarily due to genotypic variations influenced by the location of cultivation, prevailing environment, rainfall, pest and disease incidences among others [27, 43]. The selection of germplasm lines with early flowering and maturing for the development of new improved varieties will assist farmers in obtaining yield in a shorter duration, thereby accelerating production. CLP and PPP have a direct and positive influence on the PPP in adzuki bean. The economic yield of grains in legumes is directly related to PPP, and in our study, it ranged from 7.9 to 41.9. Earlier researchers also reported wide variations in PPP in adzuki bean documented between 5 to 132.5 [28], 2.0 to 109.7 [43], 22 to 122 [32], 7.67 to 116 [33], these values were higher than those observed in our study. In the current study wide variations in SPP, ranging from 5.3 to 10.0 with an average of 7.64 seeds, was observed. SPP have also been reported with varying ranges between 1.7 to 11 [43] and 4.4 to 9.8 [33]. Direct yield contributing traits, namely SYPP and SW, exhibited variations in our study, SYPP ranging from 6.36 to 47.17 g and SW ranging from 5.33 to 19.42 g. In the study conducted by Hu et al., (2022) [43], SYPP and SW were reported to range between 1.3 to 35.4 g and 1.7 to 18.5 g respectively. This difference can be attributed to the inclusion of crop wild relatives along with landraces in their study, which have significantly smaller seeds than the landraces. However, higher range of SW values have been reported earlier [28, 32, 33]. As mentioned earlier CLP, PPP, SPP and SW are major elements that determine yield. Thus, the selection of adzuki bean accessions that are relatively performing best for these traits may be important genetic sources [17, 33, 43].

In our present investigation, variability between the accessions was observed in the qualitative traits. Significant variability in qualitative traits was also reported earlier [33], and the results were similar to our work. Traits like flower colour influence pollination, stem and pod pigmentation, and biochemicals contain defence related compounds that protect from pest and disease infestation and UV radiations. Similarly, stem and pod pubescence also act as defensive tools against pests and diseases. Henceforth, variation for these traits is important in breeding for improved cultivars [17, 44, 45].

The primary goal of examining the variability and heritability of a trait is to assess the potential for effective selection [46]. In our study, the quantitative trait PPP exhibited very high values for both PCV and GCV, followed by SYPP, CLP, PPC, PB and PH. Within the group of traits with medium PCV and GCV values, the SW trait had higher values than SPP. Among the traits with the lowest values, DM has the lowest, followed by DFF. Similarly, Priya *et al*., (2021) [47] also reported high PCV and GCV values for PPP, CLP and SYPP and found low values for DFF in black gram. Heritability (H^2^) for all the traits studied we found to be high with PH and PPP being the highest value. Similar results were reported by Priya et al. (2021) [47] found high H^2^ values for all the quantitative traits studied. High GCV, PCV, and H^2^ indicate the presence of genetic variability and the potential for effective selection of these traits. GAM for the majority of the traits, including PPP, SYPP, CLP, PPC, PH, PB, SW and SPP was high. This indicates a significant presence of additive genetic variance in these traits, making selection based on them an effective and beneficial strategy. However, the GAM for DFF and DM was found to be medium, suggesting a moderate influence of environmental factors on these traits. As a result, the effectiveness of selection for these specific traits may be affected by environmental factors.

In PCA maximum variations were contributed by PPP, CLP, PB, PPC and PH. It was reported by Hu et al., (2022) [43] that 72.5% of the variability contributed by PH, PB, SW and a number of nodes in the main stem was accounted for by the first three principal components. In our study, a greater amount of genetic diversity was indicated to be present between the accessions of adzuki bean.

In Principal Component 1, the traits PPP, CLP, PB, and PPC exhibit the highest contribution to variability, making them valuable for use in future breeding programs. Conversely, the traits PH and DFF are currently underutilized and require further enhancement.

The correlation analysis of quantitative traits in adzuki bean unveiled a significant positive correlation between DFF and DM, a relationship previously observed by Hu et al., (2022) [35, 43] and Desta et al., (2023) [33]. This positive correlation suggests that an early flowering time can lead to early maturity, facilitating the development of short-duration varieties. This trait is advantageous for farmers as it allows for early pod harvesting, fetching higher prices in the market, and enables timely land preparation for subsequent crops. DM demonstrated a highly significant negative correlation with PPC in contrast to findings by Desta et al., (2023) [33] which reported a positive correlation. However, PPC exhibited positive correlations with PPP and SPP, both indicative of high yield. This underscores the potential use of identified early-maturing and high-yielding accessions in variety release or as parental lines in breeding programs, aligning with the desired traits in adzuki beans and other legumes [28, 33, 43, 48, 49]. Additionally, PH exhibited a significant positive correlation with PB, CLP, and PPP. The association between PH and PB underscores the importance of plant height in providing more internodes, facilitating increased PB production. These correlations are consistent with findings in adzuki beans and other legume crops [17, 33, 48].

CLP exhibited positive correlations with DM, SYPP, SW, and SPP, although these correlations were not statistically significant. While DFF exhibited negative and non-significant correlations with the traits PB, CLP, PPC, and PPP. This indicates that these traits have limited usefulness in terms of their correlations.

The multivariate analysis, conducted through hierarchical cluster analysis (HCA), effectively grouped 100 accessions into two major clusters. This analytical approach, as employed by previous researchers [17, 28, 30, 33, 44, 50], has proven valuable in grouping accessions and aiding in the selection of multiple desired traits within these accessions.

Out of all the available molecular markers, simple sequence repeat (SSR) markers were frequently used by earlier workers for genetic diversity assessment. This preference is attributed to their ability to detect codominance, reproducibility, distribution throughout the genomic region, polymorphism, not influence by environment, not bound to crop stage and affordability [20, 43, 51–54]. In the present study, 22 polymorphic primers generated a total of 50 alleles with an average of 2.27 bands per primer, which was comparable with earlier reports [21, 53, 55]. However, higher number of alleles per locus (5.81) have been reported by Li-xia et al. (2012) [52], during the investigation of genetic diversity of 158 core collections of adzuki bean germplasm and 12 wild relatives using 85 SSR markers. SSRs with high major allele frequency suggested that these loci were more efficient in distinguishing the inbreds. Major allele frequency varied between 0.49 to 0.98. The gene diversity spanned from a high of 0.569 (AB128100) to a low of 0.030 (VR016), with an average value of 0.186. Bhardwaj et al. (2024) [56] reported comparable findings, ranging from 0.041 to 0.499 with a mean of 0.299. The observed heterozygosity varied from 0.00 (E722-432) to 0.989 (AB128100), averaging 0.134. Aside from two primers, AB128100 and VR140, the other 20 primers exhibited very low heterozygosity values. This suggests a low probability of the studied accessions being heterozygous at the marker sites, aligning with the reduced gene diversity found in this investigation [57]. Bhardwaj et al. (2024) [56] observed heterozygosity ranging from 0.000 to 0.235 in 96 adzuki bean accessions while using 11 SSR markers. The PIC value was varied between 0.03 to 0.46 with an average of 0.16. Loci with extreme allele frequencies (either very low or very high) tend to show less genetic diversity across a population, leading to lower PIC values. Our findings align with those of Bhardwaj et al. (2024) [56], who reported PIC values between 0.0399 and 0.3733, averaging 0.241, based on an analysis of 96 germplasm accessions using 11 SSR markers. Similarly, Ning et al. (2009) [53], evaluated the genetic diversity of 366 adzuki bean core collections using 13 SSR markers and identified an average PIC of 0.561. In hierarchical cluster analysis, 100 accessions of adzuki bean were grouped into three major clusters in the neighbour joining tree. These results were consistent with reports from earlier studies [17, 21]. AMOVA of 100 accessions of adzuki bean was executed to investigate the genetic diversity distribution between and within the populations. Earlier workers also used AMOVA to calculate variations within and between the population in *Vigna* crops [17, 55]. PCoA was in conformity with the population structure and cluster analysis. Similarly, Xu et al., (2008) [58] also reported that the results of PCoA were comparable to the cluster analysis.

## Conclusion

Our investigation revealed that significant genetic variations was present in the agro-morphological traits and SSR markers in adzuki bean germplasm. High allelic diversity indicates that it has a wide genetic spectrum. Adzuki bean have huge potential to be used as the main mainstream pulse crop to become a climate-resilient, sustainable crop and to elevate malnutrition. The study identified superior performing accessions which can be further selected to release as a new variety or they can be used as a parent to develop improved varieties in the global climate change. The generated data also helps in genebank management, answering the queries. The data also help to identify gaps in the collections to include under represented or missing diversity. Thus, helps to prioritize the exploration program to collect and conserve more such germplasm otherwise may be lost over time and this loss may be sometimes irreversible.

## Supporting information

S1 Fig Field layout of adzuki bean accessions for agro-morphological characterization—augmented block design. (TIF)

S2 Fig Scree plot explaining principal component variances in terms of components. (TIF)

S3 Fig Representing variations in the leaf colour of adzuki bean A- Dark green coloured leaf (IC341960); B- Green coloured leaf (EC15648); C- Yellowish green coloured leaf (IC341962).
(TIF)

S4 Fig (a). Profile of 100 accessions revealed by primer AB128100. M- Marker (100bp); 1-72- Adzuki bean accessions. (b). Profile of 100 accessions revealed by primer AB128100. M- Marker (100bp); 73-108- Adzuki bean accessions.
(ZIP)

S5 Fig Estimation of population using LnP (D) derived delta k for k from 2 to 10. (TIF)

S1 Table List of adzuki bean accessions and their passport data used in the study. (DOCX)

S2 Table Details of qualitative characters, states, code and stage of recording observation. (DOCX)

S3 Table Mean values of 10 quantitative traits of *V*. *angularis*. (DOCX)

S4 Table States, frequencies and percentages of qualitative traits of *V*. *angularis*. (DOCX)

S5 Table Distribution pattern of accessions across genetic populations identified by population genetic structure analysis. (DOCX)

S6 Table Percentage of variation explained by the first 3 axes of PCoA by 22 SSR markers. (DOCX)
