# Supplementary figures and images for: Exploring genetic diversity of potential legume, Vigna angularis (Willd.) Ohwi and Ohashi through agro-morphological traits and SSR markers analysis
Source: PLoS One. 2024 Dec 6;19(12):e0312845. doi: 10.1371/journal.pone.0312845 (PMC11623801; doi:10.1371/journal.pone.0312845)

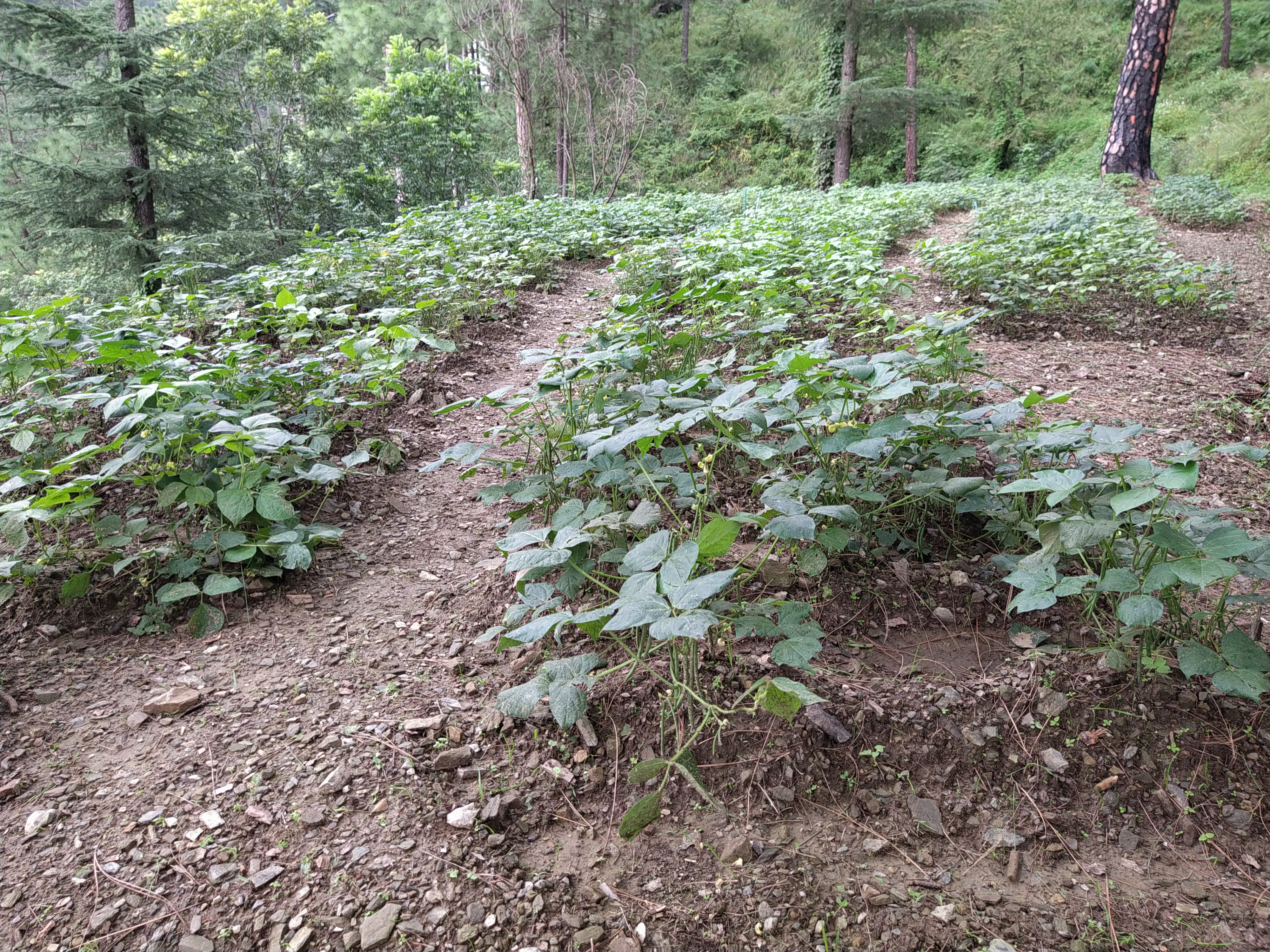

Supplement: S1 Fig — (TIF) [file pone.0312845.s001.tif]

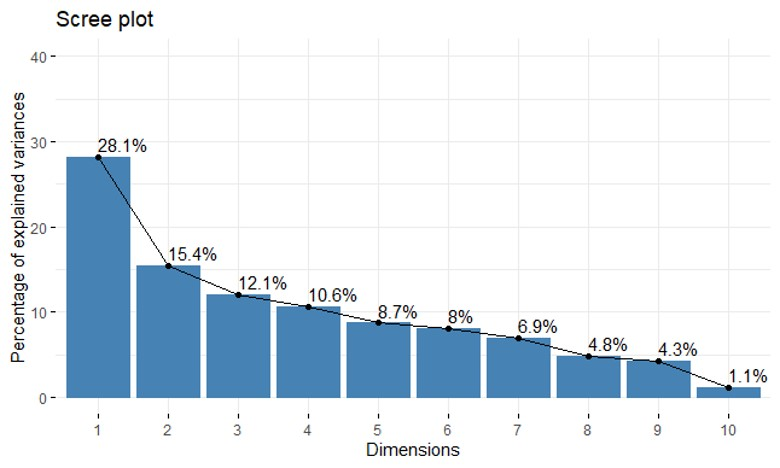

Supplement: S2 Fig — (TIF) [file pone.0312845.s002.tif]

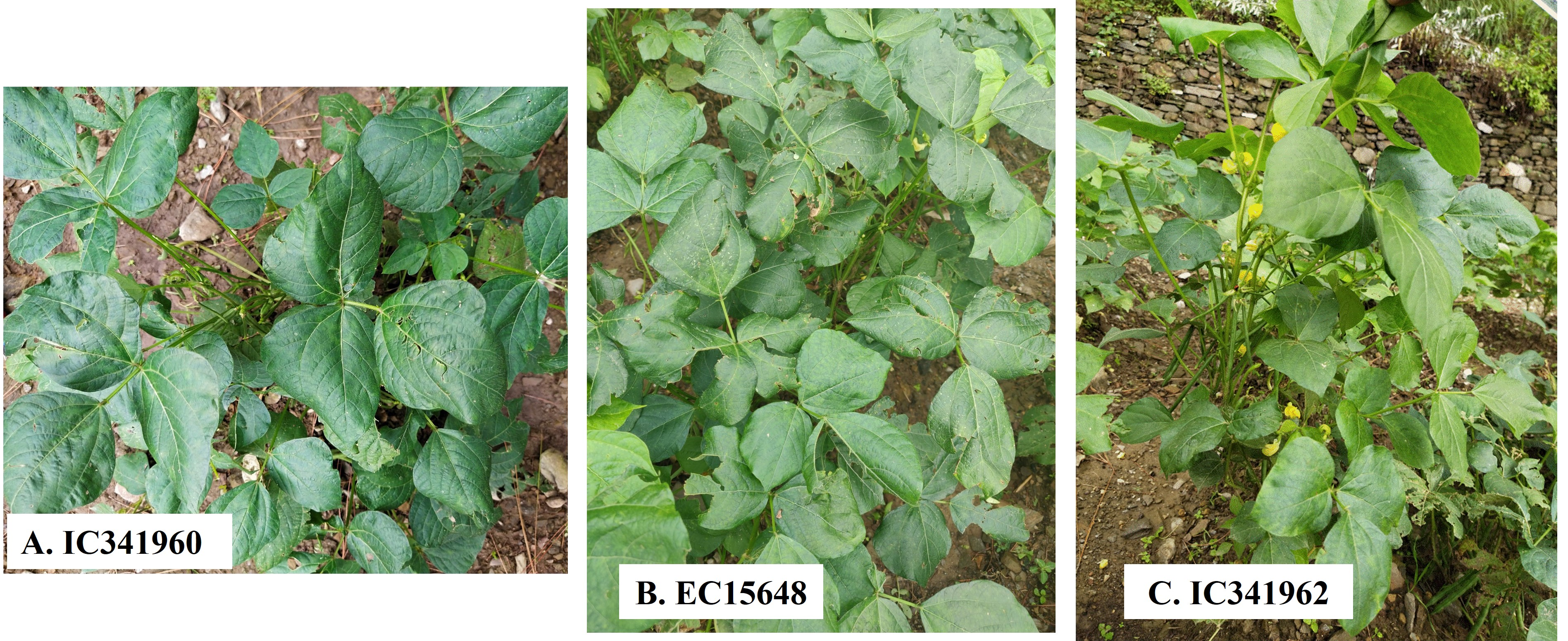

Supplement: S3 Fig — Representing variations in the leaf colour of adzuki bean A- Dark green coloured leaf (IC341960); B- Green coloured leaf (EC15648); C- Yellowish green coloured leaf (IC341962). (TIF) [file pone.0312845.s003.tif]

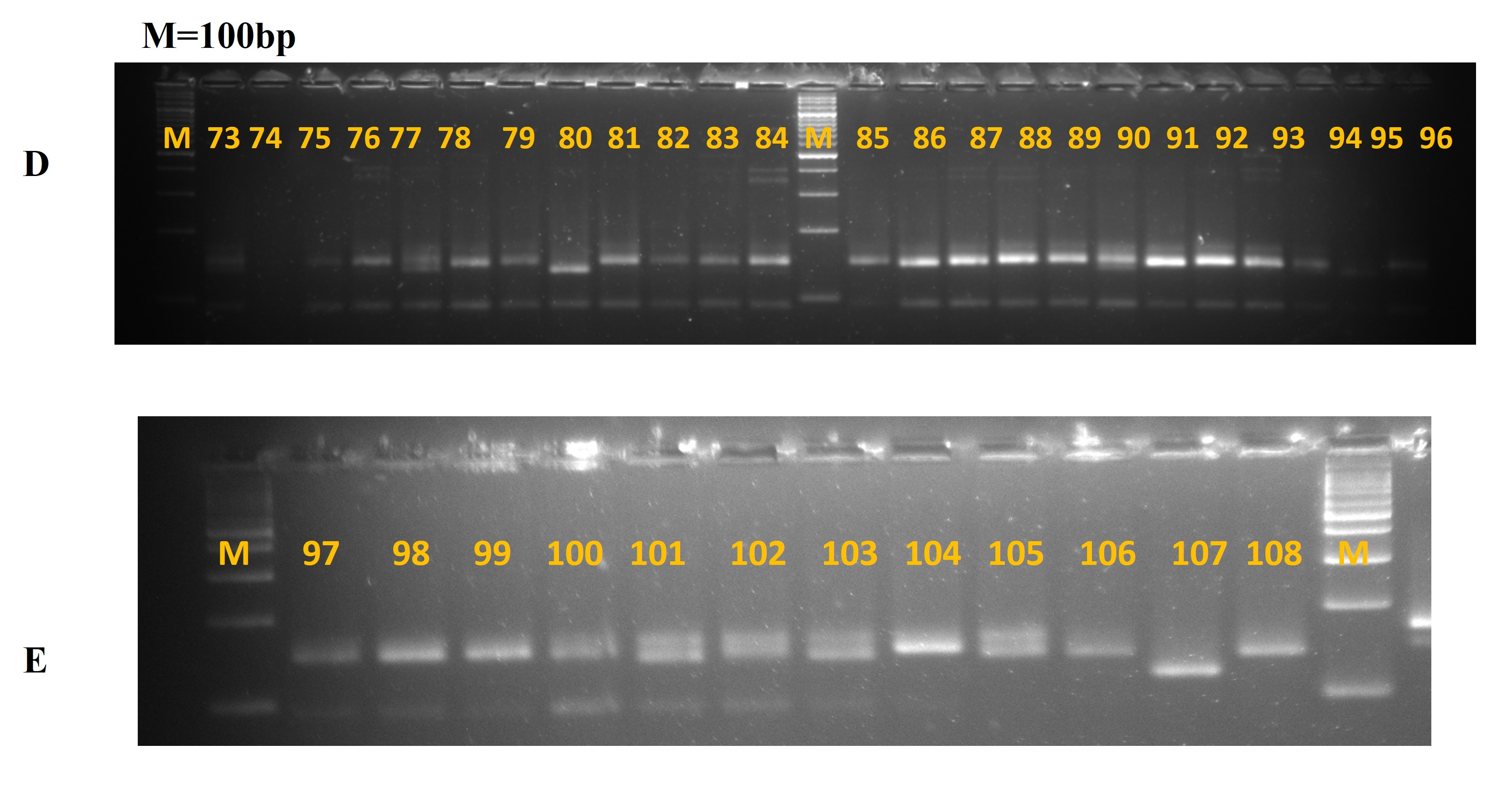

Supplement: S4 Fig — (a). Profile of 100 accessions revealed by primer AB128100. M- Marker (100bp); 1-72- Adzuki bean accessions. (b). Profile of 100 accessions revealed by primer AB128100. M- Marker (100bp); 73-108- Adzuki bean accessions. (ZIP) [file pone.0312845.s004.zip › Fig S4(b).tif]

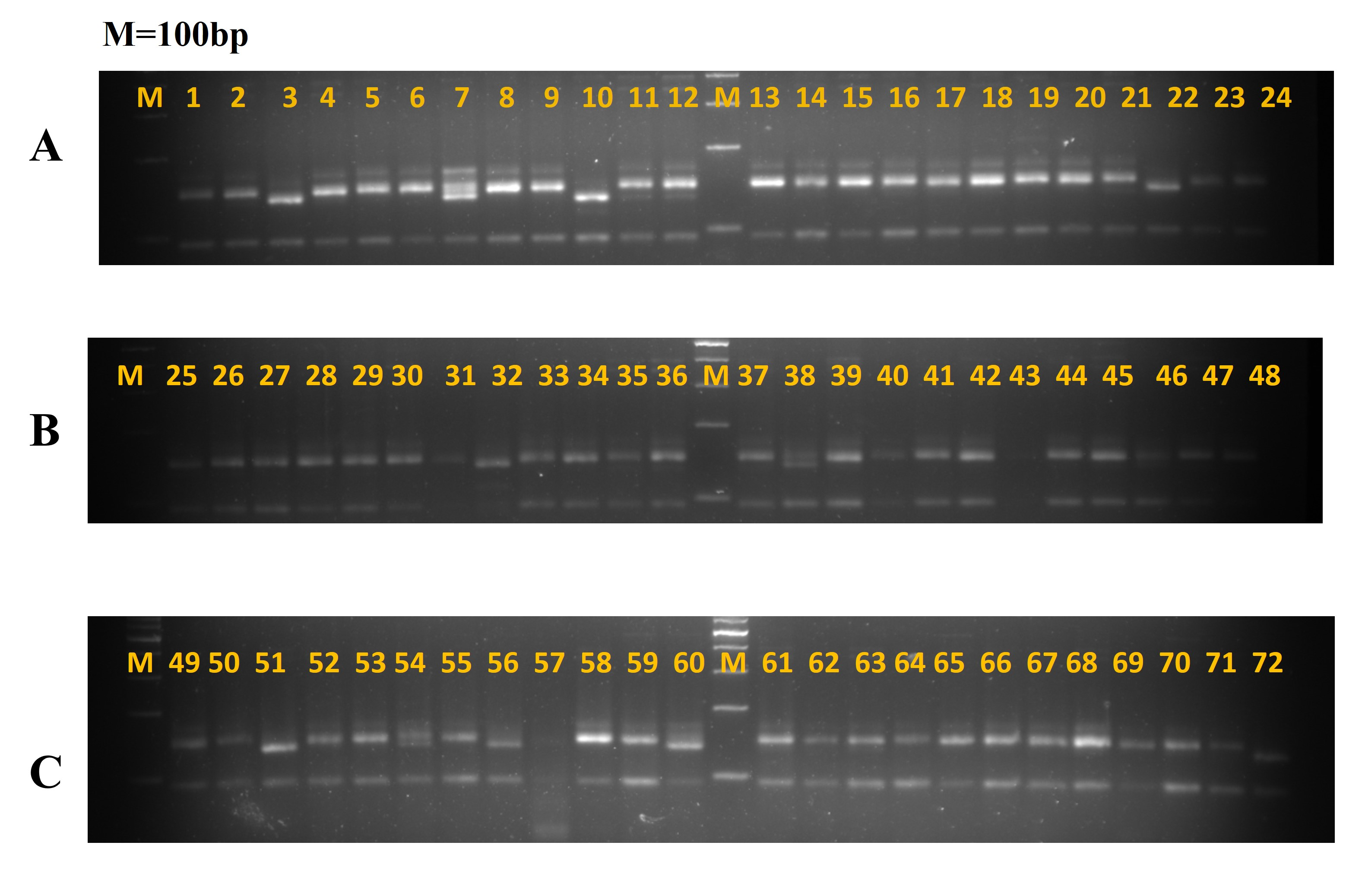

Supplement: S4 Fig — (a). Profile of 100 accessions revealed by primer AB128100. M- Marker (100bp); 1-72- Adzuki bean accessions. (b). Profile of 100 accessions revealed by primer AB128100. M- Marker (100bp); 73-108- Adzuki bean accessions. (ZIP) [file pone.0312845.s004.zip › Fig S4(a).tif]

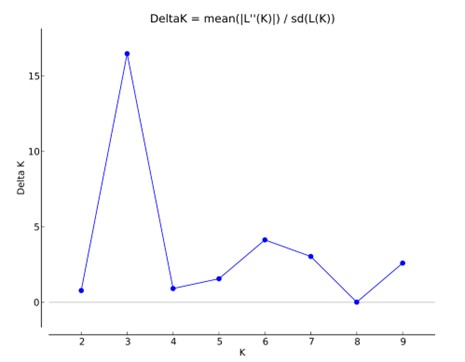

Supplement: S5 Fig — (TIF) [file pone.0312845.s005.tif]
