# Supplementary material for: Exploring genetic diversity of potential legume, Vigna angularis (Willd.) Ohwi and Ohashi through agro-morphological traits and SSR markers analysis
Source: PLoS One. 2024 Dec 6;19(12):e0312845. doi: 10.1371/journal.pone.0312845 (PMC11623801; doi:10.1371/journal.pone.0312845)
Supplement: S1 Table — (DOCX) [file pone.0312845.s006.docx]

**Table S1. List of adzuki bean accessions with their passport data used in the study**

| **No.** | **Accession number** | **Origin** |
| --- | --- | --- |
| **1** | EC000372 | Unknown |
| **2** | EC15256 | Unknown |
| **3** | EC000251 | Unknown |
| **4** | IC341938 | Himachal Pradesh |
| **5** | IC341951 | Unknown |
| **6** | IC89957 | Unknown |
| **7** | IC251353 | Unknown |
| **8** | IC341946 | Unknown |
| **9** | EC000377 | Unknown |
| **10** | EC18959 | Unknown |
| **11** | IC108556 | Unknown |
| **12** | IC341940 | Himachal Pradesh |
| **13** | EC15648 | Unknown |
| **14** | IC341948 | Unknown |
| **15** | IC140846 | Uttarakhand |
| **16** | EC18256 | Unknown |
| **17** | IC140848 | Uttarakhand |
| **18** | IC341942 | Himachal Pradesh |
| **19** | IC341950 | Himachal Pradesh |
| **20** | EC18151 | Unknown |
| **21** | IC341943 | Himachal Pradesh |
| **22** | EC000249 | Unknown |
| **23** | IC341939 | Himachal Pradesh |
| **24** | EC000248 | Unknown |
| **25** | EC18257 | Unknown |
| **26** | EC34264 | Unknown |
| **27** | EC30253 | Unknown |
| **28** | EC80850 | Unknown |
| **29** | EC36070 | Unknown |
| **30** | IC341958 | Unknown |
| **31** | IC341962 | Himachal Pradesh |
| **32** | IC341963 | Unknown |
| **33** | IC341960 | Unknown |
| **34** | IC341944 | Unknown |
| **35** | EC34027 | United States of America |
| **36** | IC469173 | Himachal Pradesh |
| **37** | IC341956 | Himachal Pradesh |
| **38** | IC341947 | Unknown |
| **39** | IC341961 | Unknown |
| **40** | EC24523 | Unknown |
| **41** | EC59459 | Unknown |
| **42** | EC57159 | Unknown |
| **43** | IC341957 | Himachal Pradesh |
| **44** | IC341952 | Himachal Pradesh |
| **45** | IC341953 | Unknown |
| **46** | IC469172 | Himachal Pradesh |
| **47** | EC24102 | Unknown |
| **48** | EC30250 | Unknown |
| **49** | EC30256 | Unknown |
| **50** | EC34625 | Unknown |
| **51** | EC59489 | Unknown |
| **52** | EC120466 | Unknown |
| **53** | IC469171 | Himachal Pradesh |
| **54** | EC281186 | Unknown |
| **55** | EC290251 | Unknown |
| **56** | EC290652 | Unknown |
| **57** | EC340240 | Unknown |
| **58** | SMLAB6 | Unknown |
| **59** | SMLAB7 | Unknown |
| **60** | SMLAB8 | Unknown |
| **61** | SMLAB9 | Unknown |
| **62** | HPKAB53 | Himachal Pradesh |
| **63** | HPKAB87 | Unknown |
| **64** | HPKAB95 | Unknown |
| **65** | HPKAB98 | Unknown |
| **66** | EC340244 | United States of America |
| **67** | EC340254 | United States of America |
| **68** | EC340257 | United States of America |
| **69** | EC340259 | United States of America |
| **70** | EC340261 | United States of America |
| **71** | EC340271 | United States of America |
| **72** | SMLAB3 | Unknown |
| **73** | SMLAB4 | Unknown |
| **74** | SMLAB5 | Unknown |
| **75** | IC341955 | Unknown |
| **76** | IC353547 | Uttarakhand |
| **77** | IC341959 | Himachal Pradesh |
| **78** | EC340263 | United States of America |
| **79** | EC120460 | USSR |
| **80** | EC340283 | United States of America |
| **81** | IC339653 | Jammu and Kashmir |
| **82** | IC341941 | Himachal Pradesh |
| **83** | EC87896 | Unknown |
| **84** | EC36973A | Unknown |
| **85** | EC057459 | Unknown |
| **86** | IC469175 | Himachal Pradesh |
| **87** | EC340250 | United States of America |
| **88** | EC000276 | Unknown |
| **89** | IC455396 | Unknown |
| **90** | EC000264 | Unknown |
| **91** | IC341954 | Unknown |
| **92** | IC469174 | Himachal Pradesh |
| **93** | IC108080 | Unknown |
| **94** | EC340251 | United States of America |
| **95** | IC485385 | Unknown |
| **96** | IC16761 | Unknown |
| **97** | IC341937 | Himachal Pradesh |
| **98** | IC341945 | Unknown |
| **99** | IC341949 | Unknown |
| **100** | EC30270 | Unknown |
| **101** | HPU-51 | Unknown |
| **102** | Totru Local | Unknown |
| **103** | Grams Local2 | Unknown |
