# Supplementary material for: Exploring genetic diversity of potential legume, Vigna angularis (Willd.) Ohwi and Ohashi through agro-morphological traits and SSR markers analysis
Source: PLoS One. 2024 Dec 6;19(12):e0312845. doi: 10.1371/journal.pone.0312845 (PMC11623801; doi:10.1371/journal.pone.0312845)
Supplement: S2 Table — (DOCX) [file pone.0312845.s007.docx]

**Table S2. Details of qualitative characters, states, code and stage of recording observation**

| **S. N.** | **Characters** | **State** | **Code** | **Observation** |
| --- | --- | --- | --- | --- |
|  | Early plant vigour | Poor  Good  Very good | 1  2  3 | Recorded after 25 days of sowing |
|  | Plant habit | Determinate  Indeterminate  Others | 1  2  99 | Recorded at full foliage stage |
|  | Plant growth habit | Erect  Spreading  Others | 1  2  99 | Recorded at full foliage stage |
|  | Leaf colour | Yellowish green  Green  Dark green  Others | 1  2  3  99 | Recorded at full foliage stage |
|  | Leaf surface | Glabrous  Pubescent  Others | 1  2  99 | Recorded at full foliage stage |
|  | Leaflet shape | Entire  Lobed  Others | 1  2  99 | Recorded at full foliage stage |
|  | Flower colour | Light yellow  Yellow  Orange  Others | 1  2  3  99 | Recorded at full blossom stage |
|  | Stem colour | Light yellow  Purple  Green  Others | 1  2  3  99 | Recorded at full blossom stage |
|  | Stem surface | Glabrous  Pubescent  Others | 1  2  99 | Recorded at full blossom stage |
|  | Pod angle | Erect  Pendent  Others | 1  2  99 | Recorded at near maturity stage |
|  | Pod surface | Glabrous  Pubescent  Others | 1  2  99 | Recorded at 50% flowering |
|  | Seed coat colour | Green  Brown  Maroon  Red  Others | 1  2  3  4  99 | Recorded immediately after harvesting |
