# Supplementary material for: Exploring genetic diversity of potential legume, Vigna angularis (Willd.) Ohwi and Ohashi through agro-morphological traits and SSR markers analysis
Source: PLoS One. 2024 Dec 6;19(12):e0312845. doi: 10.1371/journal.pone.0312845 (PMC11623801; doi:10.1371/journal.pone.0312845)
Supplement: S3 Table — (DOCX) [file pone.0312845.s008.docx]

**Table S3. Mean values of 10 quantitative traits of *V. angularis***

| **S.NO** | **ACC NO.** | **DFF** | **PB** | **CLP** | **PPC** | **PPP** | **PH**  **(cm)** | **DM** | **SYPP**  **(g)** | **SW**  **(g)** | **SPP** |
| --- | --- | --- | --- | --- | --- | --- | --- | --- | --- | --- | --- |
| **1** | EC000372 | 63 | 1.42 | 6.15 | 2.92 | 19.42 | 63.58 | 123 | 18.06 | 9.38 | 6.75 |
| **2** | EC15256 | 69 | 2.42 | 13.25 | 2.15 | 17.90 | 55.23 | 124.5 | 47.17 | 10.49 | 9.07 |
| **3** | EC000251 | 64 | 3.50 | 8.30 | 2.57 | 16.00 | 57.76 | 126 | 8.97 | 13.08 | 6.50 |
| **4** | IC341938 | 63.5 | 1.58 | 8.45 | 2.25 | 12.40 | 34.53 | 123 | 16.18 | 11.75 | 7.56 |
| **5** | IC341951 | 88 | 1.50 | 6.75 | 1.75 | 10.42 | 39.04 | 126 | 15.50 | 12.19 | 6.50 |
| **6** | IC89957 | 61 | 1.00 | 6.00 | 2.55 | 9.15 | 35.10 | 123 | 13.44 | 10.01 | 5.56 |
| **7** | IC251353 | 62 | 3.17 | 13.42 | 1.75 | 18.83 | 59.56 | 123 | 12.82 | 5.33 | 10.03 |
| **8** | IC341946 | 57 | 1.78 | 4.65 | 3.07 | 8.42 | 36.58 | 95 | 17.42 | 12.09 | 7.50 |
| **9** | EC000377 | 54.5 | 2.17 | 6.75 | 2.17 | 8.57 | 45.02 | 97 | 23.52 | 11.33 | 6.00 |
| **10** | EC18959 | 55.5 | 2.80 | 6.25 | 2.00 | 12.92 | 66.39 | 99 | 11.45 | 9.05 | 9.00 |
| **11** | IC108556 | 56.5 | 1.50 | 7.42 | 2.42 | 11.25 | 37.26 | 96 | 15.41 | 10.72 | 7.25 |
| **12** | IC341940 | 54.5 | 2.17 | 6.69 | 2.57 | 12.73 | 41.27 | 97 | 17.38 | 10.71 | 6.28 |
| **13** | EC15648 | 57 | 1.90 | 8.42 | 2.58 | 14.67 | 32.15 | 100.5 | 9.71 | 10.58 | 7.54 |
| **14** | IC341948 | 54.5 | 2.75 | 11.50 | 2.15 | 18.15 | 56.89 | 100 | 14.71 | 11.07 | 6.48 |
| **15** | IC140846 | 58 | 1.83 | 9.25 | 5.90 | 26.50 | 41.12 | 97.5 | 12.52 | 8.55 | 8.95 |
| **16** | EC18256 | 53.5 | 1.90 | 6.42 | 3.07 | 11.65 | 37.45 | 107 | 18.04 | 10.58 | 9.38 |
| **17** | IC140848 | 56.5 | 1.75 | 7.50 | 2.00 | 19.42 | 38.30 | 105.5 | 14.92 | 11.52 | 6.54 |
| **18** | IC341942 | 56 | 3.50 | 11.25 | 3.92 | 25.65 | 57.25 | 106.5 | 11.58 | 12.06 | 9.50 |
| **19** | IC341950 | 55.5 | 2.90 | 8.25 | 3.42 | 23.50 | 61.34 | 103 | 15.56 | 10.28 | 10.00 |
| **20** | EC18151 | 56 | 2.42 | 9.58 | 2.15 | 16.92 | 75.69 | 101 | 16.62 | 9.44 | 9.50 |
| **21** | IC341943 | 58.5 | 2.00 | 7.40 | 3.23 | 25.00 | 47.04 | 98 | 10.09 | 10.01 | 8.23 |
| **22** | EC000249 | 54.5 | 2.42 | 9.35 | 3.42 | 23.07 | 44.72 | 108.5 | 19.50 | 11.52 | 10.00 |
| **23** | IC341939 | 58.5 | 2.42 | 17.17 | 2.65 | 28.40 | 54.30 | 110 | 8.75 | 10.88 | 6.50 |
| **24** | EC000248 | 61 | 2.90 | 11.25 | 2.42 | 21.57 | 72.82 | 100.5 | 19.76 | 17.56 | 7.50 |
| **25** | EC18257 | 62 | 1.75 | 5.42 | 2.50 | 9.75 | 55.73 | 97 | 18.30 | 13.49 | 7.00 |
| **26** | EC34264 | 58.5 | 2.65 | 15.65 | 3.25 | 41.15 | 60.93 | 106 | 15.81 | 10.64 | 9.50 |
| **27** | EC30253 | 59 | 2.75 | 10.00 | 3.65 | 31.58 | 43.37 | 99.5 | 14.22 | 10.69 | 7.15 |
| **28** | EC80850 | 55.5 | 2.50 | 7.75 | 3.42 | 25.42 | 54.42 | 99.5 | 11.36 | 9.11 | 8.50 |
| **29** | EC36070 | 54.5 | 3.00 | 13.25 | 2.65 | 27.07 | 68.42 | 99 | 16.07 | 10.90 | 5.04 |
| **30** | IC341958 | 58 | 1.50 | 4.92 | 3.83 | 17.92 | 59.84 | 97 | 11.85 | 10.05 | 10.00 |
| **31** | IC341962 | 60.5 | 2.42 | 14.15 | 3.55 | 31.08 | 55.70 | 113.5 | 9.07 | 7.75 | 7.53 |
| **32** | IC341963 | 59.5 | 2.42 | 7.55 | 3.73 | 19.42 | 48.33 | 100.5 | 11.44 | 9.61 | 6.50 |
| **33** | IC341960 | 58.5 | 2.50 | 11.25 | 3.00 | 23.08 | 58.10 | 123 | 9.19 | 11.22 | 9.04 |
| **34** | IC341944 | 53.5 | 1.58 | 4.92 | 2.30 | 13.15 | 61.48 | 97.5 | 11.56 | 11.57 | 4.50 |
| **35** | EC34027 | 54.5 | 2.25 | 10.65 | 2.57 | 22.33 | 47.56 | 102.5 | 11.50 | 9.39 | 8.50 |
| **36** | IC469173 | 54.5 | 1.83 | 9.00 | 2.57 | 14.75 | 59.57 | 98.5 | 6.36 | 8.29 | 8.57 |
| **37** | IC341956 | 56.5 | 1.90 | 9.00 | 3.07 | 16.65 | 48.92 | 108.5 | 7.59 | 11.22 | 7.58 |
| **38** | IC341947 | 60.5 | 1.75 | 5.92 | 1.92 | 8.92 | 46.77 | 107 | 9.87 | 10.81 | 7.03 |
| **39** | IC341961 | 59 | 1.50 | 5.15 | 3.15 | 8.58 | 45.24 | 106 | 7.74 | 9.82 | 6.57 |
| **40** | EC24523 | 59.5 | 2.25 | 7.42 | 2.83 | 18.42 | 55.52 | 107 | 11.68 | 10.34 | 9.58 |
| **41** | EC59459 | 58 | 1.42 | 6.00 | 2.07 | 13.75 | 63.88 | 112.5 | 16.34 | 8.35 | 9.13 |
| **42** | EC57159 | 57.5 | 2.42 | 5.15 | 3.65 | 15.08 | 58.66 | 117 | 12.43 | 10.42 | 7.63 |
| **43** | IC341957 | 70 | 2.75 | 9.42 | 1.90 | 15.15 | 73.95 | 123 | 6.61 | 6.41 | 6.54 |
| **44** | IC341952 | 58.5 | 2.25 | 6.50 | 2.42 | 11.58 | 45.35 | 103 | 5.51 | 9.59 | 7.50 |
| **45** | IC341953 | 61.5 | 1.65 | 8.00 | 1.73 | 11.17 | 56.47 | 105 | 7.56 | 12.91 | 7.75 |
| **46** | IC469172 | 58 | 2.17 | 5.75 | 1.42 | 8.73 | 53.38 | 106 | 10.37 | 11.07 | 8.58 |
| **47** | EC24102 | 60.5 | 2.50 | 5.92 | 2.58 | 11.90 | 48.37 | 101 | 12.73 | 9.71 | 9.25 |
| **48** | EC30250 | 61 | 2.15 | 7.15 | 1.57 | 13.42 | 49.83 | 113.5 | 8.43 | 7.82 | 5.50 |
| **49** | EC30256 | 58.5 | 1.65 | 5.92 | 1.57 | 7.90 | 51.76 | 115 | 14.53 | 10.50 | 4.45 |
| **50** | EC34625 | 55.5 | 1.65 | 6.15 | 1.50 | 8.92 | 49.14 | 117 | 11.63 | 9.45 | 5.50 |
| **51** | EC59489 | 87.5 | 1.65 | 6.50 | 2.17 | 11.07 | 43.51 | 121 | 11.76 | 9.51 | 6.50 |
| **52** | EC120466 | 61 | 1.42 | 5.00 | 1.65 | 9.92 | 48.50 | 117 | 14.50 | 10.22 | 7.07 |
| **53** | IC469171 | 64 | 2.50 | 8.42 | 1.42 | 16.58 | 57.77 | 120 | 11.69 | 9.18 | 7.48 |
| **54** | EC281186 | 56.5 | 2.15 | 11.25 | 2.42 | 21.57 | 53.89 | 118 | 17.66 | 16.18 | 6.70 |
| **55** | EC290251 | 55 | 1.65 | 7.00 | 2.65 | 17.58 | 53.28 | 121.5 | 16.02 | 12.47 | 7.72 |
| **56** | EC290652 | 54.5 | 2.50 | 9.92 | 2.75 | 15.15 | 41.69 | 117 | 12.51 | 9.39 | 7.63 |
| **57** | EC340240 | 57 | 1.42 | 6.15 | 1.75 | 13.42 | 47.72 | 122.5 | 14.60 | 12.47 | 10.00 |
| **58** | SMLAB6 | 59.5 | 2.25 | 4.75 | 1.73 | 10.42 | 47.71 | 122 | 6.56 | 9.83 | 8.46 |
| **59** | SMLAB7 | 59 | 2.42 | 7.25 | 2.07 | 14.40 | 59.18 | 126 | 6.66 | 19.42 | 7.58 |
| **60** | SMLAB8 | 56.5 | 1.58 | 6.15 | 2.42 | 12.08 | 50.39 | 126.5 | 8.11 | 12.24 | 8.53 |
| **61** | SMLAB9 | 55 | 2.42 | 7.42 | 1.73 | 12.58 | 49.07 | 126 | 13.86 | 14.60 | 7.50 |
| **62** | HPKAB53 | 54.5 | 1.65 | 8.67 | 1.50 | 14.25 | 46.61 | 117 | 8.50 | 13.73 | 7.21 |
| **63** | HPKAB87 | 55.5 | 2.50 | 11.65 | 2.57 | 22.15 | 62.35 | 124.5 | 7.86 | 9.45 | 5.58 |
| **64** | HPKAB95 | 57 | 2.25 | 9.75 | 2.23 | 21.58 | 54.64 | 117 | 8.29 | 16.05 | 6.50 |
| **65** | HPKAB98 | 56 | 1.42 | 5.92 | 2.67 | 13.33 | 49.41 | 116 | 8.45 | 10.26 | 8.00 |
| **66** | EC340244 | 56.5 | 1.42 | 5.92 | 1.42 | 9.50 | 60.03 | 120.5 | 13.37 | 10.54 | 5.48 |
| **67** | EC340254 | 54.5 | 2.65 | 10.90 | 1.75 | 19.08 | 53.43 | 122.5 | 13.44 | 12.84 | 8.88 |
| **68** | EC340257 | 53.5 | 1.50 | 5.15 | 1.42 | 11.00 | 45.99 | 123 | 13.52 | 10.41 | 8.00 |
| **69** | EC340259 | 70 | 1.50 | 4.92 | 1.90 | 9.25 | 47.66 | 128.5 | 13.35 | 11.25 | 8.13 |
| **70** | EC340261 | 62 | 2.25 | 6.50 | 1.65 | 9.57 | 34.55 | 118 | 11.42 | 10.87 | 7.38 |
| **71** | EC340271 | 62.5 | 1.50 | 8.00 | 1.58 | 16.57 | 59.40 | 113 | 20.49 | 16.41 | 6.17 |
| **72** | SMLAB3 | 61.5 | 1.42 | 7.50 | 1.50 | 15.00 | 52.40 | 113.5 | 6.69 | 11.52 | 6.50 |
| **73** | SMLAB4 | 67 | 1.65 | 8.67 | 1.73 | 14.65 | 42.67 | 111 | 8.39 | 11.35 | 7.63 |
| **74** | SMLAB5 | 55 | 1.65 | 8.65 | 1.58 | 14.40 | 54.59 | 109.5 | 11.44 | 10.19 | 6.33 |
| **75** | IC341955 | 60 | 1.65 | 6.42 | 1.75 | 10.50 | 52.31 | 125 | 11.44 | 12.04 | 10.00 |
| **76** | IC353547 | 61 | 1.50 | 6.00 | 2.00 | 10.42 | 43.28 | 116.5 | 8.59 | 10.28 | 9.50 |
| **77** | IC341959 | 62.5 | 1.50 | 7.65 | 3.07 | 12.65 | 50.64 | 110 | 10.53 | 9.43 | 7.70 |
| **78** | EC340263 | 61 | 2.42 | 10.73 | 1.73 | 11.92 | 44.07 | 106 | 11.77 | 10.42 | 8.50 |
| **79** | EC120460 | 67 | 1.42 | 7.94 | 2.42 | 22.23 | 47.54 | 90 | 20.92 | 11.63 | 9.38 |
| **80** | EC340283 | 53.5 | 1.00 | 4.67 | 2.42 | 10.92 | 28.99 | 93.5 | 20.43 | 10.57 | 7.63 |
| **81** | IC339653 | 58 | 2.17 | 8.15 | 2.65 | 17.08 | 57.82 | 107 | 16.26 | 11.73 | 9.45 |
| **82** | IC341941 | 56.5 | 2.17 | 8.50 | 2.75 | 14.15 | 32.82 | 91 | 9.58 | 8.27 | 6.13 |
| **83** | EC87896 | 66 | 1.42 | 6.00 | 2.57 | 11.57 | 28.31 | 102 | 8.76 | 13.04 | 6.83 |
| **84** | EC36973A | 65 | 1.42 | 10.50 | 3.92 | 26.08 | 77.75 | 105.5 | 11.78 | 8.63 | 6.50 |
| **85** | EC057459 | 62.5 | 1.70 | 9.00 | 3.40 | 30.40 | 76.15 | 100 | 19.57 | 12.34 | 8.54 |
| **86** | IC469175 | 69.5 | 3.65 | 12.00 | 3.65 | 41.92 | 70.83 | 118.5 | 8.50 | 9.80 | 8.53 |
| **87** | EC340250 | 72.5 | 1.33 | 8.40 | 3.33 | 20.58 | 65.56 | 115 | 11.47 | 14.09 | 9.50 |
| **88** | EC000276 | 57.5 | 2.50 | 13.92 | 3.65 | 36.15 | 58.73 | 108.5 | 19.00 | 12.95 | 6.50 |
| **89** | IC455396 | 57 | 1.58 | 10.08 | 3.67 | 35.42 | 61.43 | 112.5 | 12.66 | 12.32 | 9.92 |
| **90** | EC000264 | 57.5 | 2.65 | 18.42 | 2.65 | 37.67 | 50.06 | 110 | 9.51 | 10.43 | 7.41 |
| **91** | IC341954 | 57.5 | 4.00 | 8.42 | 2.58 | 22.15 | 61.80 | 116 | 8.37 | 10.32 | 6.58 |
| **92** | IC469174 | 58 | 3.17 | 7.75 | 2.90 | 24.08 | 95.45 | 113.5 | 8.60 | 14.76 | 6.53 |
| **93** | IC108080 | 60 | 3.25 | 10.40 | 2.42 | 25.73 | 70.97 | 110.5 | 13.29 | 12.32 | 7.74 |
| **94** | EC340251 | 62.5 | 1.90 | 6.67 | 3.40 | 20.25 | 59.63 | 103.5 | 9.12 | 11.32 | 7.75 |
| **95** | IC485385 | 69.5 | 2.42 | 8.65 | 1.75 | 18.57 | 77.65 | 114.5 | 8.90 | 9.67 | 6.50 |
| **96** | IC16761 | 62 | 2.90 | 16.92 | 3.42 | 34.58 | 63.95 | 107 | 18.72 | 12.07 | 9.50 |
| **97** | IC341937 | 59 | 2.17 | 15.65 | 3.57 | 34.50 | 67.73 | 113.5 | 11.89 | 16.02 | 7.50 |
| **98** | IC341945 | 60 | 3.75 | 7.00 | 3.33 | 20.00 | 49.79 | 112 | 10.74 | 10.08 | 6.33 |
| **99** | IC341949 | 63 | 3.42 | 9.67 | 2.65 | 21.00 | 73.54 | 103 | 8.22 | 11.01 | 6.33 |
| **100** | EC30270 | 62.5 | 3.25 | 10.67 | 2.00 | 20.42 | 84.27 | 115.5 | 16.62 | 10.24 | 9.74 |
| **C1** | HPU-51 | 56.3 | 2.52 | 11.89 | 2.91 | 29.60 | 56.11 | 102.9 | 13.39 | 12.95 | 6.76 |
| **C2** | Totru Local | 53.2 | 2.77 | 11.64 | 3.05 | 33.28 | 51.77 | 113.5 | 9.49 | 5.79 | 7.49 |
| **C3** | Grams Local 2 | 59.9 | 2.08 | 15.29 | 3.17 | 34.04 | 65.83 | 115.0 | 12.48 | 15.37 | 7.43 |

**DFF**: Days to 50% flowering, **PB**: Primary branches, **CLP**: Number of clusters per plant, **PPC**: Number of pods per plant, **PH**: Plant height, **DM**: Days to 80% of maturity, **SYPP**: Seed yield per plant, **SW**: Seed weight, **SPP**: Number of seeds per pod, **C1**, **C2** and **C3**: Check1, 2 and 3
