# Supplementary material for: Exploring genetic diversity of potential legume, Vigna angularis (Willd.) Ohwi and Ohashi through agro-morphological traits and SSR markers analysis
Source: PLoS One. 2024 Dec 6;19(12):e0312845. doi: 10.1371/journal.pone.0312845 (PMC11623801; doi:10.1371/journal.pone.0312845)
Supplement: S4 Table — (DOCX) [file pone.0312845.s009.docx]

##### Table S4. States, frequencies and percentages of qualitative traits of *V. angularis*

| **Qualitative traits** | **State** | **Frequencies** | **Percent %** |
| --- | --- | --- | --- |
| Early plant vigour | Poor | 10 | 10.0 |
|  | Good | 77 | 77.0 |
|  | Very good | 13 | 13.0 |
| Plant growth habit | Erect | 91 | 91.0 |
|  | Spreading | 0 | 0.00 |
|  | Others | 9 | 9.00 |
| Leaf colour | Yellowish green | 4 | 4.00 |
|  | Green | 49 | 49.0 |
|  | Dark green | 47 | 47.0 |
| Stem colour | Light yellow | 0 | 0.00 |
|  | Purple | 3 | 3.00 |
|  | Green | 97 | 97.0 |
| Seed coat colour | Green | 0 | 0.00 |
|  | Black mottle | 8 | 8.00 |
|  | Yellow | 4 | 4.00 |
|  | Red | 87 | 87.0 |
|  | Others | 1 | 1.00 |
