# Supplementary material for: Exploring genetic diversity of potential legume, Vigna angularis (Willd.) Ohwi and Ohashi through agro-morphological traits and SSR markers analysis
Source: PLoS One. 2024 Dec 6;19(12):e0312845. doi: 10.1371/journal.pone.0312845 (PMC11623801; doi:10.1371/journal.pone.0312845)
Supplement: S5 Table — (DOCX) [file pone.0312845.s010.docx]

**Table S5. Distribution pattern of accessions across genetic populations identified by population genetic structure analysis**

| **Population** | **Pure individuals** | **Admixture** | **Total** |
| --- | --- | --- | --- |
| Population Ⅰ | 26 | 16 | 42 |
| Population Ⅱ | 15 | 13 | 28 |
| Population Ⅲ | 20 | 10 | 30 |
