# Supplementary material for: Exploring genetic diversity of potential legume, Vigna angularis (Willd.) Ohwi and Ohashi through agro-morphological traits and SSR markers analysis
Source: PLoS One. 2024 Dec 6;19(12):e0312845. doi: 10.1371/journal.pone.0312845 (PMC11623801; doi:10.1371/journal.pone.0312845)
Supplement: S6 Table — (DOCX) [file pone.0312845.s011.docx]

**Table S6. Percentage of variation explained by the first 3 axes of PCoA by 22 SSR markers**

| **Axis** | **% Variance** | **Cumulative Variance** |
| --- | --- | --- |
| **1** | 11.59 | 11.59 |
| **2** | 9.43 | 21.02 |
| **3** | 8.06 | 29.07 |
